# Ratiometric Mechano-Fluorescent Elastomers Dually Promoted via Effective Force-Triggered Radicals and Preeminent Toughnesses/Stretchabilities by Unconventional Shuttling Dimensions of Tetraphenylethylene-Suspended [c2] Daisy Chain Rotaxanes

**DOI:** 10.1021/acsmaterialsau.5c00182

**Published:** 2025-11-04

**Authors:** Tu Thi Kim Cuc, Ting-Chi Wu, Pham Quoc Nhien, Trang Manh Khang, Shunmuga Nathan Shunmuga Nainar, Bui Thi Buu Hue, Wei-Tsung Chuang, Hsiu-Hui Chen, Michal Kohout, Hong-Cheu Lin

**Affiliations:** † Department of Materials Science and Engineering, 34914 National Yang Ming Chiao Tung University, Hsinchu 300093, Taiwan; ‡ Department of Chemistry, College of Natural Sciences, Can Tho University, Can Tho City 94000, Viet Nam; § National Synchrotron Radiation Research Center, Hsinchu 300092, Taiwan; ∥ Department of Molecular Science and Engineering, 34877 National Taipei University of Technology, Taipei 106335, Taiwan; ⊥ Department of Organic Chemistry, University of Chemistry and Technology Prague, Prague 16628, Czech Republic; # Center for Emergent Functional Matter Science, National Yang Ming Chiao Tung University, Hsinchu 300093, Taiwan

**Keywords:** tetraarylsuccinonitrile mechanophores, daisy chain molecules, artificial molecular muscle tougheners, polyurethane elastomers, ratiometric fluorescent responses

## Abstract

Innovative radical-type mechano-fluorescent polyurethane (PU) elastomers were developed by integrating axle- and macrocycle-exerted force modes of tetraphenylethylene (TPE)-functionalized daisy chain rotaxanes with expanded/contracted conformations into polymer matrices, revealing distinct mechanical and optical performances under external tensile forces. Surprisingly, the designed PU films containing negligible amounts (0.02% molar ratio of all monomers) of daisy chain structures with unconventional shuttling dimensions (as artificial molecular muscle tougheners) exhibited ultrastretchable capabilities and preeminent toughnesses, possessing a record-high toughness of 1363 MJ/m^3^ at the strain rate of 20 mm/s, approximately 6.3 times tougher than the standard PU films, along with a significant loading weight ratio of 150,000. Additionally, appealing ratiometric fluorescent responses between blue-emissive TPE stoppers and yellow-emissive diarylacetonitrile radical species could be detected in PU films by stretching due to the introduction of TPE-based daisy chain rotaxanes into mechano-fluorophoric PU skeletons, enabling reversible dual fluorescent switching during tensile loading and unloading processes. Remarkably, notable shape memory and reversible ratiometric fluorescence behaviors of synthetic daisy chain-grafted PU films could be accessible by thermal treatments, indicating probable applications of mechanically interlocked molecule-functionalized PU films with splendid mechanical and optical features for designing stimuli-responsive smart materials.

## Graphical abstract

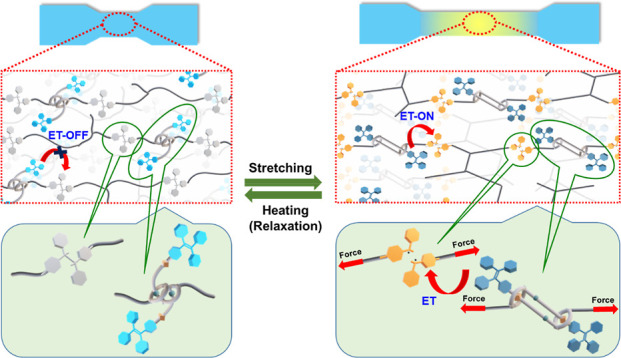

## Introduction

Smart materials are recognized as a particular type of stimuli-responsive materials, which can visibly, tangibly, and reversibly respond to various kinds of electrical, chemical, mechanical, thermal, magnetic triggers, etc., and permit the alterations of their physicochemical characteristics.^1–4^ So far, the innovation of stimuli-responsive fluorescent materials, whose notably fluorescent emission variations can be acquired by using a wide assortment of external stimuli, has become essentially important in current multidisciplinary chemistry from the perspective of multifunctional materials owing to their greatly sensitive responses and facile visualizations.^5–15^ Significantly, the emergences of stimuli-responsive fluorescent materials possessing aggregation-induced emission (AIE) features along with switchable fluorescent emissions have enabled the developments of advanced materials with high-performance versatile functions to be potentially applied to numerous fields from physical chemistry to materials science, biotechnology, and pharmacotherapy.^16–25^

To date, molecular machines derived from mechanically interlocked architectures have been widely developed to fabricate stimuli-responsive molecular systems since the existences and the relative internal movements of the macrocyclic components in mechanically interlocked molecules (MIMs) can be adjusted by a quantity of diverse stimuli to exhibit dramatic modifications of their intrinsic chemical, physical, and electronic properties.^26–36^ Importantly, mechanically interlocked daisy chain rotaxanes, a representative class of MIMs, have appeared as promising functionalized molecular units to build muscle-like materials, which can be attributed to their unique capabilities of stimuli-controlled molecular muscle-like contraction/expansion motions by applying external triggers.^37–50^ Accordingly, the integrations of functionalized daisy chain moieties with slidable components into polymeric platforms acting as molecular muscles have been broadly explored to provide outstanding features that will not be achievable through traditional covalent linkages for constructing smart and modern polymeric materials with potentially controllable and switchable behaviors.^51–57^

Appealingly, mechanical forces are regarded as one type of external stimulations for mechanochromic polymeric materials possessing mechanically caused photophysical changes, which expose great prospective in the applications of fluorescence switches, mechano-sensors, memory devices, security systems, etc.^58–61^ In particular, mechanophores are force-active molecular species in response to external mechanical stimuli by suffering expectable structural rearrangements and chemical transformations, which are frequently exploited to be conjugated into a variety of polymer matrices for the foundation of mechanochromic polymers. Additionally, a range of developed mechano-responsive molecules have been mainly categorized into ring opening- (isomerization-) and radical formation-based mechanophores.^62–76^ Being a peculiar and an intriguing group of stimuli-responsive polymeric materials, shape memory polymers (SMPs), which are highly deformable polymers and can memorize their original shapes, have been gaining growing attention from the aspects of both fundamental research studies and industrial inventions.^77–79^ Notably, polyurethanes (PUs) have become the most talented candidates among SMPs due to their exclusive properties, i.e., highly modifiable structures, admirable mechanical properties, large recoverable deformations, low glass-transition temperatures, etc.^80,81^ Currently, PUs commonly serve as promising polymer skeletons for producing miscellaneous mechano-sensitive PUs decorated with specified mechanophores to be recommended in the development of intelligent and advanced materials.^82–87^

Benefiting from the force-driven molecular shuttling functions of threaded structures, MIM-based rotaxanes possessing quencher–fluorophore pairs have been employed as mechanochromic fluorescent force transducers to be implanted into PU scaffolds to generate rotaxane-based mechanophoric polymers, which have been proclaimed lately.^88–91^ Interestingly, it is noteworthy that a minor amount (ca. 1.0 wt %) of the synthetic fluorophoric polyrotaxane with slidable cyclic molecules was incorporated into PU polymer networks containing mechanochromic rhodamine derivatives to obtain ultrastretchable polyrotaxane-based PU films with ratiometric fluorescent emissions toward mechanical forces.^92^ In addition, the extended and contracted conformations of fluorophoric [c2] daisy chain molecules were embedded into mechano-sensitive PU frameworks to acquire excellent mechanical and optical characteristics upon tensile loading, which have been proposed recently.^93^ Crucially, the intrinsic stretchabilities and toughnesses of the designed PU films comprising small amounts (ca. 0.03% molar amounts of all monomers) of daisy chain rotaxanes with long-range slipping movements in PU backbones could be clearly boosted.

In an effort to offer state of the art MIM-based polymeric materials with stimuli-responsive behaviors, a variety of mechanical force-induced mechano-fluorescent MIM-functionalized PU elastomers embracing radical-type tetraarylsuccinonitrile (**TASN**) mechano-fluorophores and [c2] daisy chain molecules in expanded/contracted conformations with different shuttling dimensions of axle- and macrocycle-exerted force modes as artificial molecular muscles have been constructed productively. In particular, these aimed mechanically interlocked [c2] daisy chain rotaxanes with different shuttling dimensions involve tetraphenylethylene (TPE) derivatives acting as bulky capped groups and AIE-fluorophoric energy donors to achieve the ratiometric fluorescent responses via energy transfer (ET) processes, which were covalently implanted into the backbone of radical-type mechano-fluorescent PU elastomers to progress their innate mechanical and photophysical performances. Additionally, the reversible conversions between the contracted and expanded states of AIE-fluorophoric [c2] daisy chain rotaxanes can be manipulated by adding external chemical stimuli to attain dissimilar conformations of [c2] daisy chain molecules, allowing the distinctive features of corresponding PU elastomers to be obtained. Intriguingly, the yellow-emitting diarylacetonitrile (**DAAN** ^•^) radical species can be generated by the cleavages of original nonemitting **TASN** moieties in PU films upon mechanical forces through the covalent bond breakage mechanisms to offer the stable colored radicals, leading to the occurrence of ET-ON processes between blue-emitting TPE donors and yellow-emitting **DAAN** ^•^ acceptors to gain dual fluorescent emission changes in PU elastomers. Besides, the shape memory performances along with fluorescent emission retrievals of stretched PU films can also be inspected through thermal treatments to further confirm their real-world applications for the beginning of specific kinds of modern materials and devices.

## Results and Discussion

### Molecular Design and Synthesis

According to previous publications,^82,93^ AIE-fluorophoric [c2] daisy chain rotaxanes **DTS/E** in its expanded form and **DTM/C** in its contracted form with various shuttling dimensions of axle- and macrocycle-exerted force modes were designed and synthesized as shown in Schemes S3 and S5. Moreover, the molecular shuttling motions of DB24C8 macrocycles from dibenzylammonium (DBA) to *N*-methyltriazolium stations would emerge to offer different [c2] daisy chain molecules of traditional **DTS/C** in the contracted form and unconventional **DTM/E** in the expanded form by the neutralizations of DBA binding sites in the corresponding [c2] daisy chain rotaxanes **DTS/E** and **DTM/C** (see Scheme S3 and S5 along with Figure 1). As a result, it was suggested that the synthetic [c2] daisy chain molecules possessed the reversible molecular shuttling of expanded **DTS/E** and **DTM/E** together with contracted **DTS/C** and **DTM/C** conformations because of the reciprocating movements of DB24C8 rings between two disparate recognition stations toward external chemical stimulations (see Figure 1 and Movies S1 and S2). Afterward, the decoupled [c2] daisy chain rotaxanes, i.e., **DTS/E** and **DTS/C** units containing axle-exerted force modes along with **DTM/C** and **DTM/E** moieties holding macrocycle-exerted force modes, were covalently incorporated into radical-type mechano-fluorophoric PU skeletons consisting of **TASN** derivatives to produce **PU-TASN-DTS/E**, **PU-TASN-DTS/C**, **PU-TASN-DTM/E**, and **PU-TASN-DTM/C** polymeric elastomers, respectively. It could be assumed that decoupled [c2] daisy chain molecules would disclose different responses in PU films under tensile forces owing to their distinctive molecular structural natures with different shuttling dimensions of axle- and macrocycle-exerted force modes. Specifically, daisy chain moieties in **PU-TASN-DTS/E** and **PU-TASN-DTM/E** films would mostly tighten their compact conformations along with constrained sliding motions, whereas those in **PU-TASN-DTS/C** and **PU-TASN-DTM/C** films would undergo prominent gliding movements and then tighten their conformations similar to the others in **PU-TASN-DTS/E** and **PU-TASN-DTM/E** films. The abbreviations used for daisy chain molecules and PU films are defined in Table S1.

**Figure 1:**
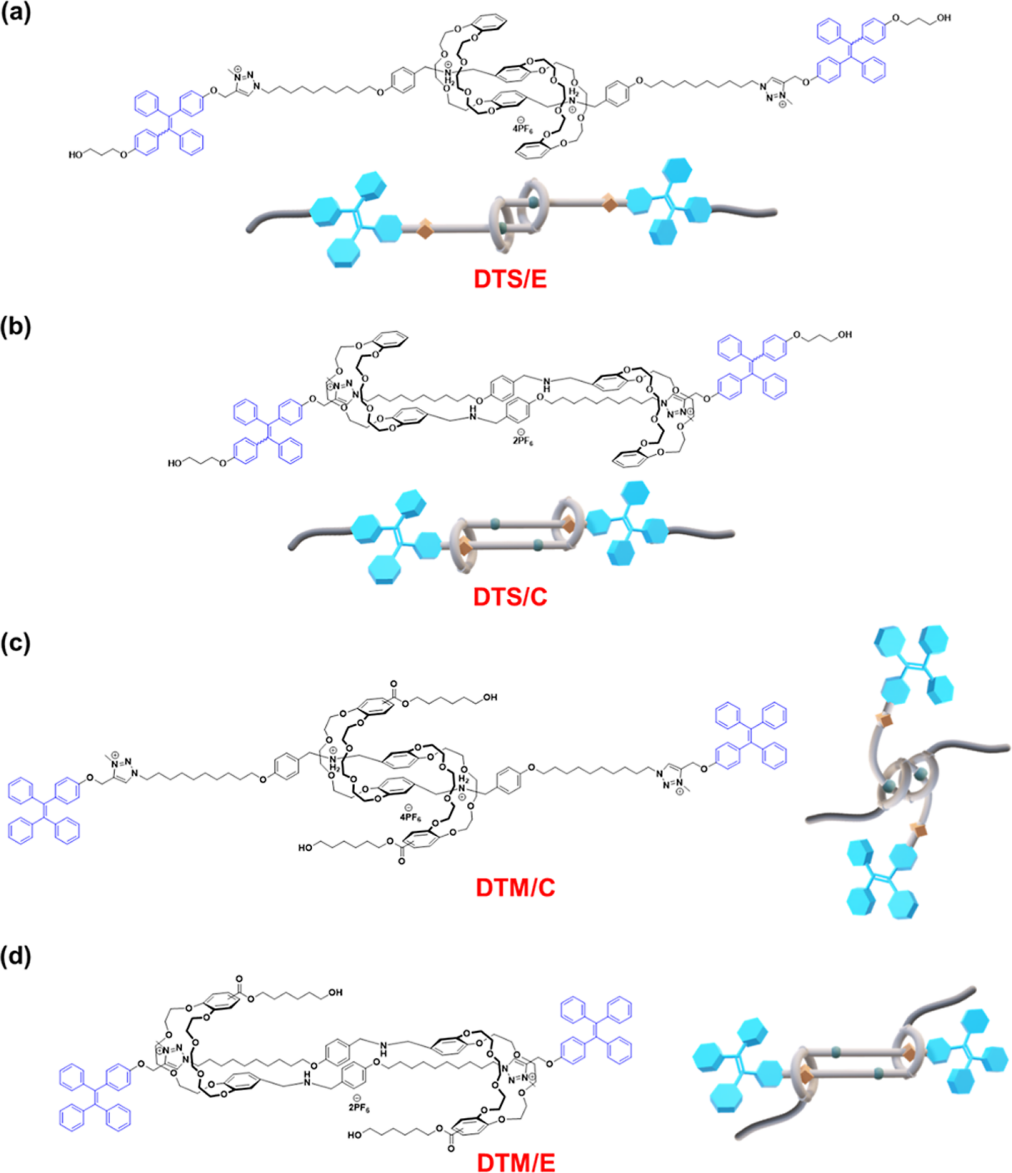
Chemical structures and cartoon representations of various shuttling dimensions of [c2] daisy chain rotaxanes, i.e., traditional (a) **DTS/E** and (b) **DTS/C** along with unconventional (c) **DTM/C** and (d) **DTM/E**, where **E** and **C** are denoted as expanded and contracted forms, respectively.

The synthetic approaches of aimed mechano-fluorescent PU elastomers with the incorporations of varied expanded/contracted [c2] daisy chains possessing different shuttling dimensions of axle- and macrocycle-exerted force modes are shown in Figure 2, where these mechano-sensitive PUs consisted of diverse amounts of **TASN** derivative, hexamethylene diisocyanate (HDI), tetraethylene glycol (TEG), triethanolamine (TEA), and [c2] daisy chain units. The completely synthetic procedures and related chemical characterizations of all intermediates and targeted compounds are listed in the Supporting Information. The step-growth polymerizations were employed to create the mechano-sensitive elastomeric PUs containing **TASN** units, which were stimulated by dibutyltin dilaurate (DBTDL) with HDI as the monomer, TEG as the chain extender, TEA as the cross-linker, **TASN** derivative as the mechano-fluorophore, and various shuttling dimensions of [c2] daisy chains as molecular artificial muscles (see Figure 2 and Movie S3). On the other hand, the control sample **PU-TASN-TPE** was also delivered without [c2] daisy chain structures imbedded in polymer matrices for a judgment (see Figure 2).

**Figure 2:**
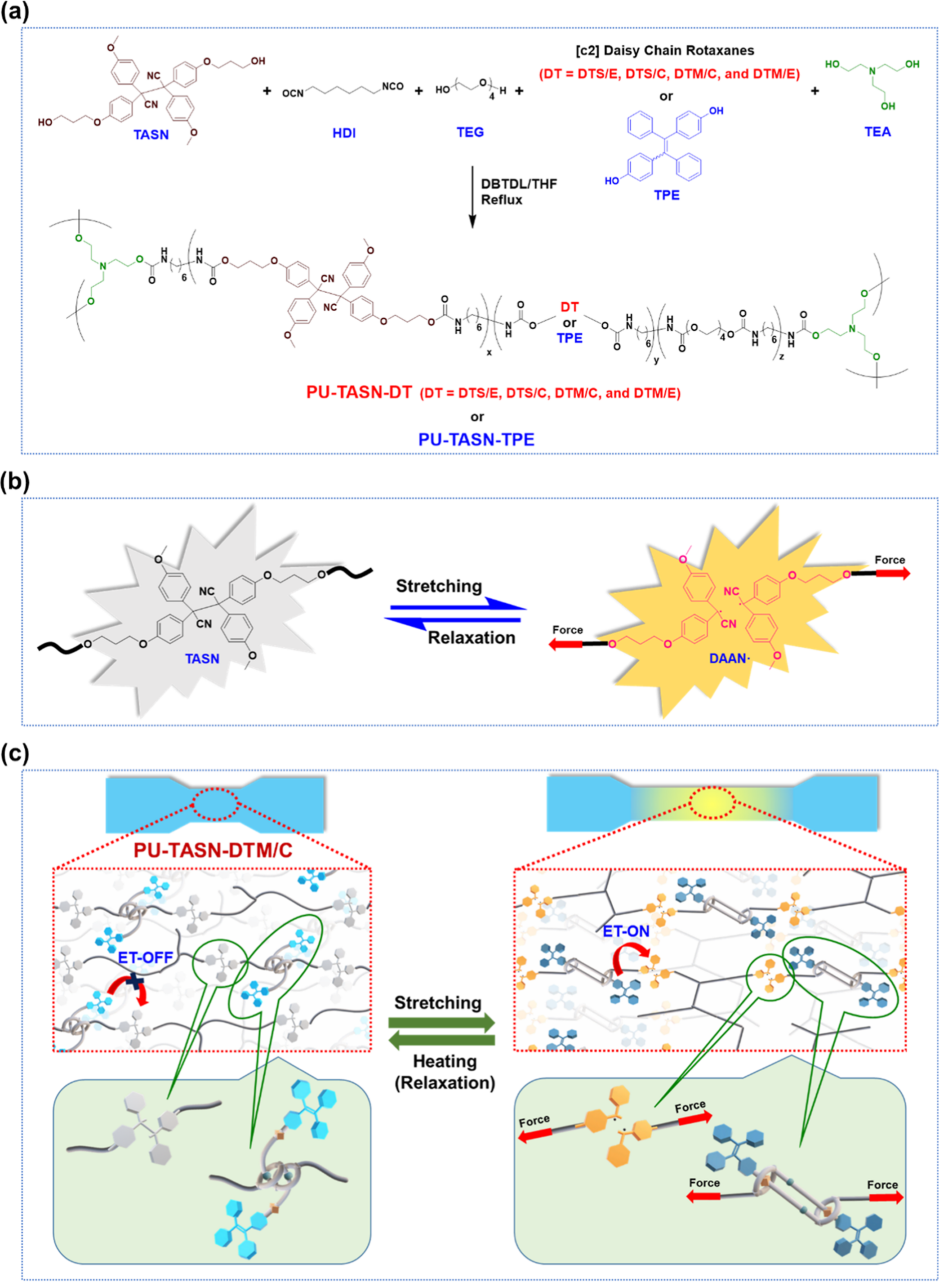
(a) Synthetic routes and chemical structures of mechano-fluorescent PU elastomers containing **TASN** mechano-fluorophore and various [c2] daisy chain rotaxanes (i.e., **DTS/E**, **DTS/C**, **DTM/C**, and **DTM/E**) along with the control sample **PU-TASN-TPE**. (b) Schematic illustration of the force-induced fluorescent emission from the nonemissive **TASN** unit to yellow-emissive **DAAN** ^**•**^ species (i.e., before and after stretching of PU films, respectively). (c) Cartoon representations of mechano-fluorophoric **PU-TASN-DTM/C** film before and after stretching.

### Chemical and Thermal Characterizations of PU Films

In order to investigate the reactions between –NCO (in HDI) and –OH groups (in TEG, TEA, **TASN**, **TPE**, and daisy chain moieties) for the productions of amide units in PU films upon poly condensation reactions (see Figure 2), FTIR measurements have been carried out. As shown in Figure S1a, the stretching vibration of –OH groups in TEG and TEA could be noticed around 3300 cm^–1^, and the characteristic band at 2250 cm^–1^ that relates to –NCO groups in HDI could also be perceived. Nevertheless, the broad band around 3300 cm^–1^ became narrower, and the typical band at 2250 cm^–1^ vanished, along with the new N–H stretching vibration that arose at 3300 cm^–1^ after polymerization. In addition, the emergence of amide I and II bands at 1685 and 1520 cm^–1^, respectively, specified that the reaction between the –NCO and –OH groups almost ended. The amide I band corresponded to C=O stretching vibrations, whereas the amide II band resulted from a combination of N–H bending and C–N stretching vibrations. Moreover, the thermal features of prepared PU films could be confirmed by thermogravimetric analysis (TGA) in Figure S1b, where TGA curves revealed highly thermal stabilities of PU elastomers up to 250 °C.

### Mechanical Properties of PU Films

The superb mechanical strengths along with excellent stretchable capabilities were acknowledged as the most important factors of PU elastomers for their practical applications in mechano-fluorophoric stretchable sensors to distinguish fluorescent emission changes upon tensile loading. Therefore, the feed molar ratios of the components were summarized and are shown in Table S2 to identify optimal stoichiometric proportions for the fabrication of PU films with acceptable mechanical performances. The molar amount of **TASN** mechano-fluorophore was fixed at 0.05 mmol (1.0 equiv), and then ca. 0.15 mmol (3.0 equiv) of TEA was added in the reaction system to facilitate the formation of a **PU-TASN-TPE** film with a medium tensile stress of 13.96 MPa and a tensile strain ca. 1835%, which was further exploited to survey the boosted mechanical toughness derived from the introductions of daisy chain components with different shuttling dimensions of axle- and macrocycle-exerted force modes (i.e., **DTS/E**, **DTS/C**, **DTM/C**, and **DTM/E**) into PU backbones (see Figure S2). Furthermore, by using less amounts of cross-linker TEA in the range of 0–0.1 mmol (0–2.0 equiv) for **PU-TASN-TPE-1**, **PU-TASN-TPE-2**, and **PU-TASN-TPE-3** films, PU films exhibited poor mechanical properties, whereas higher contents of cross-linker TEA also resulted in **PU-TASN-TPE-4** and **PU-TASN-TPE-5** films with worse toughnesses in contrast to **PU-TASN-TPE** film. Besides, as shown in Figure S2b, the optimum amount of **TASN** mechano-fluorophore was determined ca. 0.05 mmol (1.0 equiv) because the molar ratios of **TASN** units were amplified from 0.05 (1.0 equiv) to 0.075–0.1 mmol (1.5–2.0 equiv) in corresponding **PU-TASN-TPE-7** and **PU-TASN-TPE-8** films, which enabled fragile PU films with much reduced toughnesses and stretchabilities to be produced in contrast to the **PU-TASN-TPE** film. As illustrated in Figure S2c, the ideal molar ratio of the TPE unit was found to be 0.005 mmol (0.1 equiv) to yield **PU-TASN-TPE** elastomers with tolerable (i.e., medium) mechanical properties. Remarkably, various strain rates of 5, 10, and 20 mm/s were used to explore their effects on mechanical characteristics of the **PU-TASN-TPE** film as displayed in Figure S2d, in which the shorter tensile strains were acquired with the faster strain rates. Thus, the highest strain rate of 20 mm/s (or 1200 mm/min) in comparison with 1 mm/s of our previous publications^92,93^ was designated to obtain the shortest tensile strain, which would be suitable for the further improvements of toughnesses (i.e., with both highly promoted tensile stresses and strains) upon the integrations of various [c2] daisy chain units into PU skeletons.

Consequently, to acquire PU films with excellent mechanical performances, the molar ratios of AIE-fluorophoric [c2] daisy chain rotaxanes with different shuttling dimensions of axle- and macrocycle-exerted force modes (i.e., **DTS/C** and **DTM/C**) implanted into PU frameworks were monitored and plotted in stress–strain curves of relevant PU films with a strain rate of 20 mm/s (or 1200 mm/min) (see Figure S3a,b and Tables S3 and S4). By enhancing molar amounts of [c2] daisy chain units from 0 to 0.0025 mmol (0–0.05 equiv) (see Figure S3a,b), the gradually boosted toughnesses with better stretchabilities could be detected, which might be assigned to the sliding motions of [c2] daisy chains as artificial molecular muscles to provide superior elongations. Nonetheless, as the inserted amounts of [c2] daisy chain components exceeded 0.0025 mmol (0.05 equiv), the yield stresses continuously improved, while the tensile strains obviously dropped as proven in Figure S3a,b and Tables S3 and S4. The diminished toughnesses of PU films might be attributed to the complicated and huge molecular architectures of AIE-fluorophoric [c2] daisy chain rotaxanes to enable stiffnesses and fragilities of PU films.

Appealingly, taking advantage of diverse sliding distances for AIE-active [c2] daisy chain molecules with axle- and macrocycle-exerted force modes, i.e., traditional **DTS/E** and **DTS/C** along with unconventional **DTM/E** and **DTM/C**, both **PU-TASN-DTS/C** and **PU-TASN-DTM/C** elastomers comprising daisy chains **DTS/C** and **DTM/C** with evident long-range gliding motions demonstrated the obviously greater stretchabilities and toughnesses than those of **PU-TASN-DTS/E** and **PU-TASN-DTM/E** films involving respective daisy chains **DTS/E** and **DTM/E** with smaller sliding distances as shown in Figure 3a, recommending the vital influences of muscle-like MIMs on the upgrading of inborn mechanical performances of PU elastomers. More importantly, the **PU-TASN-DTM/C** film consisting of **DTM/C** with the macrocycle-exerted force mode owned the higher toughness and stretchability than that of the **PU-TASN-DTS/C** film containing **DTS/C** with the axle-exerted force mode (see Figure 3a). As a consequence, these optimum results of the preeminent toughness (with both highest tensile stress and tensile strain) of the **PU-TASN-DTM/C** film in Figure 3a might be ascribed to the unconventional shuttling dimension of the macrocycle-exerted force mode in daisy chain **DTM/C**. This shuttling dimension involved possible complex π–π stacking or aggregations of dangling TPE stoppers through different sliding processes, unlike the traditional shuttling dimension of the axle-exerted force mode in daisy chain **DTS/C**, wherein the **PU-TASN-DTS/C** film disclosed the less restricted sliding motions. In the **PU-TASN-DTM/C** film, the constrained movements of entangled TPE stoppers behaved similarly to a capable rotaxane actuator, which could activate the release of cargo molecules appended to its axle as proposed in a recent report.^94^ In the meanwhile, owing to the overlap of shorter double axles in daisy chain **DTM/E** possessing a higher rigidity and a less stretchability in the extended form after the chemical treatment (i.e., base condition) rather than the nonoverlap of longer extended double axles of daisy chain **DTS/E** (see Figure 1a,d along with the final stages of Movies S1 and S2), the **PU-TASN-DTM/E** film in Figure 3a features a higher tensile stress but a lower tensile strain in contrast to the **PU-TASN-DTS/E** film. Accordingly, the optimal molar ratios in Table S4 were utilized to construct PU elastomers possessing applicable mechanical features with supreme tensile strain and rough stress values (ca. 5181% and 33.42 MPa, respectively), which were exploited as an optimum formula to be further studied in subsequent experiments. Initiated from their outstanding stretchabilities, the toughnesses of PU elastomers could be valued and are shown in Figure 3b and Table 1. Noticeably, the **PU-TASN-DTM/C** film revealed a preeminent toughness of 1363 MJ/m^3^ at the strain rate of 20 mm/s (or 1200 mm/min), which was almost 6.3 times higher toughness than that of 215 MJ/m^3^ witnessed in the **PU-TASN-TPE** film (without daisy chains). This significant enhancement of the toughness verified the critical task of unconventional [c2] daisy chain components (just by adding **DTM/C**/TEG = 0.05 equiv/110 equiv = 0.045% diol molar ratio of daisy chain **DTM/C**) in the augmented toughnesses of designed PU elastomers. Simultaneously, compared with the **PU-TASN-TPE** film (without daisy chains), the **PU-TASN-DTS/C** film exhibited an enhanced toughness of 836 MJ/m^3^ (ca. 3.9 times higher toughness). Accordingly, the optimal **PU-TASN-DTM/C** film displayed a preeminent toughness of 1363 MJ/m^3^ as the highest toughness value of PU elastomers to the best of our knowledge (compared with the toughness values of most recently reported PU elastomers as shown in Table S6), which was ca. 1.6 times tougher than 836 MJ/m^3^ of the **PU-TASN-DTS/C** film due to their different shuttling dimensions of macrocycle- and axle-exerted force modes, respectively.

**Figure 3:**
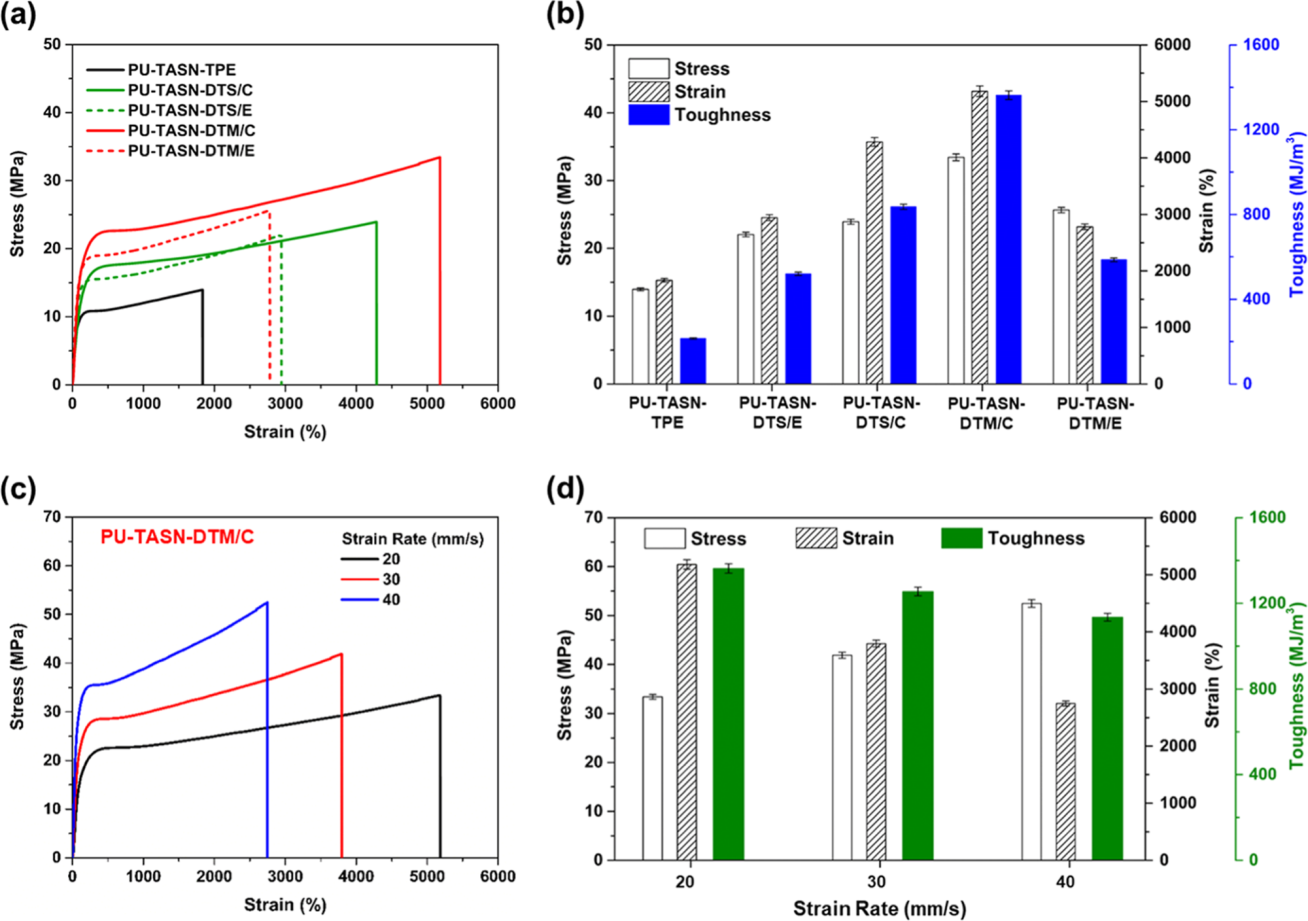
(a) Stress–strain curves and (b) stress/strain/toughness histograms of **PU-TASN-TPE**, **PU-TASN-DTS/E**, **PU-TASN-DTS/C**, **PU-TASN-DTM/C**, and **PU-TASN-DTM/E** films under tensile loading at the strain rate of 20 mm/s (or 1200 mm/min). (c) Stress–strain curves and (d) stress/strain/toughness histograms of the **PU-TASN-DTM/C** film with various strain rates (i.e., 20, 30, and 40 mm/s).

**Table 1:** Mechanical Properties of PU Films Consisting of Different Types of [c2] Daisy Chain Rotaxane Molecules

PU samples^a^	tensile stress (MPa)	tensile strain (%)	toughness (MJ/m^3^)
PU-TASN-TPE	13.96 ± 0.26	1835 ± 29	215 ± 3
PU-TASN-DTS/E	22.03 ± 0.40	2945 ± 53	520 ± 9
PU-TASN-DTS/C	23.92 ± 0.45	4285 ± 73	836 ± 14
PU-TASN-DTM/C	33.42 ± 0.63	5181 ± 84	1363 ± 22
PU-TASN-DTM/E	25.65 ± 0.48	2782 ± 50	586 ± 11

a Mechanical tests of PU films were conducted by using the strain rate of 20 mm/s (or 1200 mm/min).

In addition, as presented in Figure S2a and Table S2, among standard PU films (without any daisy chain molecules), the **PU-TASN-TPE-3** film possessed a moderate tensile stress of 12.04 MPa along with a tensile strain of ca. 1085%, which was also employed to further demonstrate the heightened mechanical features based on the integrations of daisy chain molecules (ca. 0.045% molar ratio of diol monomers) with diverse shuttling dimensions into PU frameworks. As expected, the **PU-TASN-DTM/C-3** film in Figure S3c and Table S7 exhibited an excellent toughness of 723 MJ/m^3^ accompanied by ca. 6.6 times higher toughness than that of 110 MJ/m^3^ obtained in the **PU-TASN-TPE-3** film (without daisy chains), further confirming the crucial contribution of unconventional [c2] daisy chain units in the amplified toughness of targeted PU films. In the meantime, as shown in Figure S3c and Table S7, the **PU-TASN-DTS/C-3** film also revealed an improved toughness of 433 MJ/m^3^ (ca. 3.9 times higher toughness) compared to the **PU-TASN-TPE-3** film (without daisy chains). Consequently, the **PU-TASN-DTM/C-3** film exposed an excellent toughness of 723 MJ/m^3^, which was ca. 1.7 times tougher than 433 MJ/m^3^ of the **PU-TASN-DTS/C-3** film because of their dissimilar shuttling dimensions of macrocycle- and axle-exerted force modes, respectively. Due to the unbreakable samples under tensile tests at lower strain rates of 50–100 mm/min normally reported in the listed references, the promoted values better than this record-high toughness of 1363 MJ/m^3^ should be accessible at lower strain rates than 20 mm/s (or 1200 mm/min) utilized in this study. Particularly, a preeminent toughness of 1363 MJ/m^3^ for the **PU-TASN-DTM/C** film as well as an excellent toughness of 723 MJ/m^3^ for the **PU-TASN-DTM/C-3** film could be derived from their respective stress–strain curves with the strain rate of 20 mm/s (or 1200 mm/min), and this strain rate value was much higher compared with recent publications (see Table S6). It could be noted that the enhancement of strain rates might cause higher brittleness to bring about earlier ruptures and diminished toughnesses of PU materials, which could be validated in stress–strain curves of **PU-TASN-TPE**, **PU-TASN-DTM/C**, and **PU-TASN-DTM/C-3** films with different strain rates as shown Figures S2d and S3d, and 3c,d along with their toughnesses as presented in Tables S8, S9 and 2, respectively. Owing to our instrumental limitation, tensile trains could be measured only up to 5500%, and thus, the high strain rate of 20 mm/s (or 1200 mm/min) was selected to carry out mechanical tests to yield breakages of test specimens and definite toughness values. In spite of the fact that the toughness of the optimum **PU-TASN-DTM/C** film (which should be higher than our reported value of 1363 MJ/m^3^) was sacrificed by using the high strain rate of 20 mm/s (or 1200 mm/min), its toughness value of 1363 MJ/m^3^ was still prominent in comparison with newly proposed PU films in the literature (see Table S6). Notably, the prepared **PU-TASN-DTM/C** film (weight of 20 mg) could lift a bottle weighing 3.0 kg (see Figure 4e and Movie S4), which was 150,000 times its own weight and superior to those of most lately announced PU elastomers (see Table S10), indicating its superb toughness and stretchability.

**Table 2:** Mechanical Properties of the **PU-TASN-DTM/C** Film with Different Strain Rates

strain rate (mm/s)	tensile stress (MPa)	tensile strain (%)	toughness (MJ/m^3^)
20	33.42 ± 0.63	5181 ± 84	1363 ± 22
30	41.89 ± 0.75	3794 ± 65	1255 ± 22
40	52.47 ± 0.92	2748 ± 48	1135 ± 20

**Figure 4:**
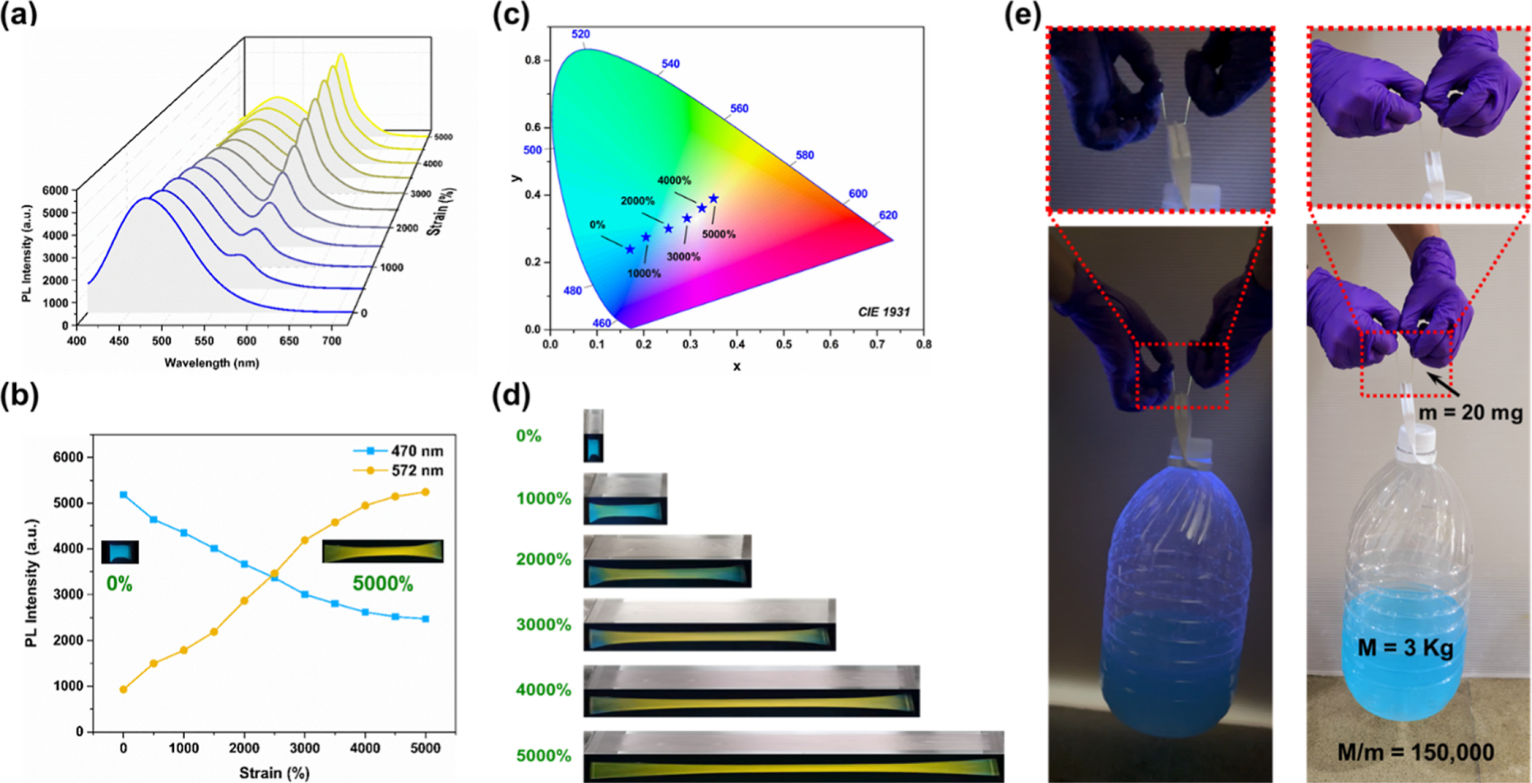
(a) PL spectra and (b) relative PL intensities of blue-emissive TPE (λ_em_ = 470 nm) and yellow-emissive **DAAN** ^**•**^ (λ_em_ = 572 nm) for the **PU-TASN-DTM/C** film with different strains. (c) CIE 1931 coordinates of the **PU-TASN-DTM/C** film with various strains up to 5000% (λ_ex_ = 365 nm). (d) Photographs of the **PU-TASN-DTM/C** film under different elongation tests, where top images were taken under ambient light and bottom images were taken under a UV lamp (λ = 365 nm). (e) Photographs of the **PU-TASN-DTM/C** film (*m* = 20 mg) lifting a plastic bottle (*M* = 3.0 kg) with a loading weight ratio of 150,000 (*M*/*m* = 150,000), where the left image was taken under a UV lamp (λ = 365 nm) and the right image was taken under ambient light.

### Mechano-Fluorescent Properties of PU Films

Essentially, the mechanical force-induced homolytic C–C bond cleavages of **TASN** derivatives as mechano-fluorophores generated stable carbon-centered radicals (**DAAN** ^**•**^) with pink colors and yellow fluorescent emissions, which have been extensively introduced into diverse polymer structures to fabricate mechanochromic polymers in recent years. Moreover, the major goals for the incorporations of AIE-fluorophoric [c2] daisy chain rotaxanes with diverse shuttling dimensions of axle- and macrocycle-exerted force modes, i.e., **DTS/E**, **DTS/C**, **DTM/C**, and **DTM/E**, to polymer networks were not only to promote intrinsic mechanical performances of PU films but also to recognize dual fluorescent emission variations through ET processes between donor–acceptor pairs, which could be mechanically managed by tensile forces. The probable schematic illustration of ratiometric fluorescent responses and possible ET processes regulated by mechanical forces for PU films involving blue-emitting TPE-based [c2] daisy chains (energy donors) and yellow-emitting mechano-fluorophoric **DAAN** ^**•**^ species (energy acceptors) is illustrated in Figure 2 and Movie S3. Therefore, the fluorescent emission alterations of **PU-TASN-DTS/C** and **PU-TASN-DTM/C** films containing TPE units and **TASN** mechano-fluorophores upon stretching were methodically examined by using PL measurements. The experimental setup for PL tests of stretched PU films was performed according to our previous study,^93^ wherein a homemade setup of a stretching ruler with scale was used to sustain elongating shapes of stretched PU films. As displayed in Figures S4 and 4, the unstretched **PU-TASN-DTS/C** and **PU-TASN-DTM/C** films revealed blue fluorescent emissions at 470 nm correlated to the existence of TPE moieties along with urethane linkages in PU films while a new peak appeared at 572 nm with yellow emission color originated from diarylacetonitrile (**DAAN** ^**•**^) radicals. The emission intensities of **DAAN** ^**•**^ radicals were intensified constantly upon stretching (see Figures S4 and 4a,b), which demonstrated that external mechanical forces could be conveyed through polymer backbones to inserted **TASN** units and triggered C–C bond breakage reactions efficiently (see Figure 2b) to cause fluorescent emission changes in both **PU-TASN-DTS/C** and **PU-TASN-DTM/C** films. Moreover, as shown in Figures S4 and 4a,b, the broad blue emission bands of TPE units and urethane linkages at 470 nm for **PU-TASN-DTS/C** and **PU-TASN-DTM/C** films were reduced gradually, whereas their mechano-fluorescent yellow emission bands of **DAAN** ^**•**^ radicals at 572 nm were heightened simultaneously upon tensile forces. The attenuations of blue fluorescent emissions belonged to TPE moieties and urethane linkages could be owing to the appearances of ET processes between blue-emitting TPE donors and yellow-emitting **DAAN** ^**•**^ acceptors accompanied by the disaggregation of TPE species in thinner films after stretching. Additionally, based on stress–strain curves of PU elastomers in Figure 3a, the **PU-TASN-DTM/C** film presented the strongest yellow fluorescent emission of **DAAN** ^**•**^ radicals at 572 nm at the longest tensile strain (ca. 5000%) due to the high degree of chemical transformations of **TASN** units. Hence, the brightest yellow **DAAN** ^**•**^ emission emerged at ca. 5000% strain with the utmost depletion of ca. 52.3% blue TPE emission intensity, and an ET efficiency of 27.8% could also be estimated by the following time-resolved photoluminescence (TRPL) experiments. Besides, the mechano-fluorescent emission switching of the **PU-TASN-DTM/C** film was noted with dynamic strains up to 5000% and signified in Movie S5 of the Supporting Information. Concurrently, the CIE diagram of the **PU-TASN-DTM/C** film in Figure 4c displayed fluorescent emission variations from blue to yellow colors with various strains. As well, photographs of the **PU-TASN-DTM/C** film under various tensile strains are afforded in Figure 4d.

Furthermore, the ultraviolet–visible light (UV–vis) spectra of **PU-TASN-DTS/C** and **PU-TASN-DTM/C** films before and after stretching were monitored and are displayed in Figure S5 to prove absorption spectral changes of TPE and **DAAN** ^**•**^ moieties. As exhibited in Figures S5a,b, the new absorption bands ca. 550 nm were ascribed to the stable carbon-centered **DAAN** ^**•**^ radicals in **PU-TASN-DTS/C** and **PU-TASN-DTM/C** films after stretching, wherein the visible color changes of PU films from quite transparent to pinkish color could be detected with the naked eye. Critically, the absorption spectrum (λ_abs_ = 550 nm) of **DAAN** ^**•**^ radicals in a stretched **PU-TASN-DTM/C** film was partly overlapped with the blue emission spectrum (λ_em_ = 470 nm) of TPE moieties in an unstretched **PU-TASN-DTM/C** film (see Figure S6a), supporting the ET processes of the released emission energy from TPE donors to be absorbed by **DAAN** ^**•**^ acceptors in the **PU-TASN-DTM/C** film upon tensile stresses. Accordingly, to certify the occurrence of ET processes from TPE donors to **DAAN** ^**•**^ acceptors, the TRPL spectra and fluorescence lifetime values accompanied by ET efficiencies of **PU-TASN-DTS/C** and **PU-TASN-DTM/C** films before and after stretching could be considered. As proven in Figure S6b, the stretched **PU-TASN-DTS/C** and **PU-TASN-DTM/C** films owned shorter lifetime values (τ_DA_ = 3.06 and 2.99 ns after ET) of TPE donors than those of unstretched **PU-TASN-DTS/C** and **PU-TASN-DTM/C** films (τ_D_ = 4.20 and 4.14 ns without ET), indicating that ET processes between TPE donors and **DAAN** ^**•**^ acceptors occurred in **PU-TASN-DTS/C** and **PU-TASN-DTM/C** films after stretching. Subsequently, by utilizing the equation *E* = 1 – (τ_DA_/τ_D_), where τ_DA_ and τ_D_ represent the lifetime values of the stretched and unstretched **PU-TASN-DTS/C** and **PU-TASN-DTM/C** films, respectively, the ET efficiencies (*E*, %) of stretched **PU-TASN-DTS/C** and **PU-TASN-DTM/C** films could be estimated to be 27.1 and 27.8%, exhibiting the attractive ratiometric fluorescent responses of AIE-fluorophoric [c2] daisy chain rotaxanes installed in mechano-sensitive polymeric platforms.

In consequence, PL spectra of the stretched **PU-TASN-DTM/C** film at ambient temperature with different time periods were scanned and are exhibited in Figure 5 to explore reversible ratiometric fluorescent switching behaviors through different discolorations of mechano-fluorescent emissions with respect to various time intervals in stretched PU films. As shown in Figures 5a,b, after the removal of tensile forces, yellow fluorescence emissions of **DAAN** ^**•**^ moieties at 572 nm decayed steadily at room temperature to reach an equilibrium blue emission after 12 h. Simultaneously, blue fluorescence emissions of TPE derivatives (λ_em_ = 470 nm) were regained progressively up to 67.8% recovery after 12 h. However, the incomplete retrieval of the blue fluorescent emission at 470 nm was incompatible with the conspicuous reduction of the yellow mechano-fluorescence at 572 nm, probably caused by the decreased thickness of elongated fragments, resulting in the unfeasibility of entire fluorescent emission restorations. On the other hand, the yellow fluorescent emission of the stretched **PU-TASN-DTM/C** film dissipated quickly by dipping into a hot water bath of ca. 70 °C directly, and the original blue fluorescent emission could be restored within 5 min, identifying the proficiently reversible mechano-fluorescent alterations between **DAAN** ^**•**^ species and **TASN** units in the designed PU films (see Figures 5c,d). Moreover, to inspect ratiometric fluorescent responses of the stretched **PU-TASN-DTM/C** film at different heating processes, PL measurements could also be employed. As exhibited in Figure S7a,b, the **PU-TASN-DTM/C** film stretched up to 5000% strain (at 25 °C) could be immersed in water at diverse temperatures (30, 40, 50, 60, and 70 °C) to survey the relaxations of yellow fluorescent emissions for **DAAN** ^**•**^ species (λ_em_ = 572 nm) accompanied by the simultaneous retrievals of blue fluorescent emissions for TPE units (λ_em_ = 470 nm). In the beginning, a slight decline of the yellow **DAAN** ^**•**^ emission (λ_em_ = 572 nm) along with a minor regaining of the blue TPE emission (λ_em_ = 470 nm) emerged at the temperature treatment of 30 °C. Later on, the major reductions of yellow **DAAN** ^**•**^ emissions along with the chief repossessions of blue TPE emissions arose at temperatures of 40, 50, and 60 °C. Once the temperature reached 70 °C, both minified yellow **DAAN** ^**•**^ and recovered blue TPE emissions were nearly saturated, signifying that the thermal treatment at 70 °C were effective enough for the stretched **PU-TASN-DTM/C** film to partially regain original blue TPE emissions before stretching, while yellow mechano-fluorescent emissions of **DAAN** ^**•**^ moieties in the stretched **PU-TASN-DTM/C** film were quite stable to display interesting mechano-fluorophoric characteristics at ambient environments for upcoming applications. In addition, the capable reversibility could be asserted by monitoring PL spectra of the **PU-TASN-DTM/C** film under several stretching–heating cycles as shown in Figure S7c,d, implying that force-responsive PU elastomers disclosed allowable reversible stabilities after five stretching–heating cycles with tiny decays of fluorescent emission intensities.

**Figure 5:**
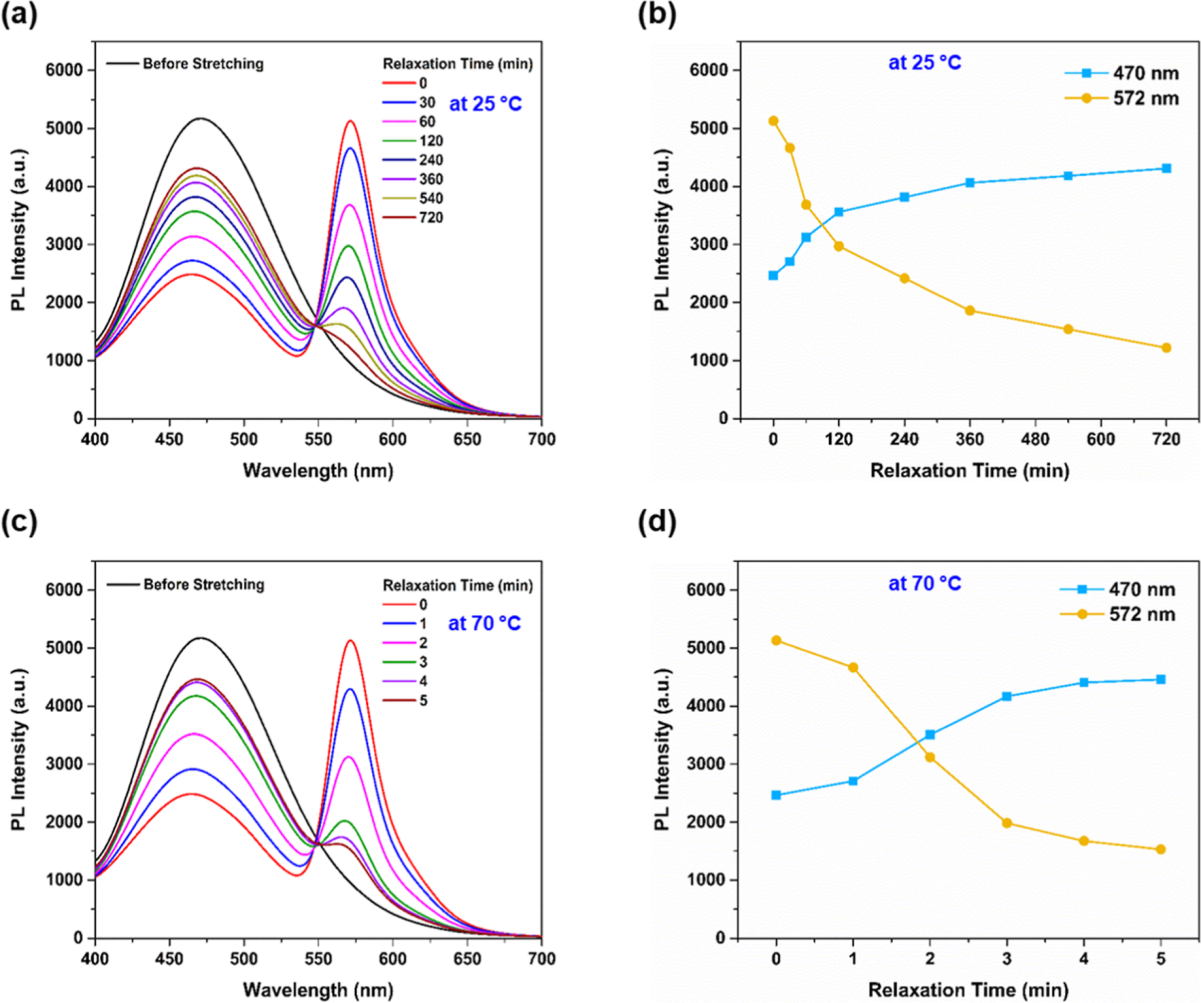
PL spectra and relative PL intensities of blue-emissive TPE (λ_em_ = 470 nm) and yellow-emissive **DAAN** ^**•**^ units (λ_em_ = 572 nm) for **PU-TASN-DTM/C** films with different relaxation time periods after stretching (a,b) at room temperature and (c,d) upon heating at 70 °C (λ_ex_ = 365 nm).

### X-ray Diffraction Analyses

Aside from the macroscopic mechanical tests, small and wide-angle X-ray scattering (SAXS and WAXS) analyses in Figure S8a–d, respectively, were also considered to study the microstructure of the **PU-TASN-DTM/C** film in elongation tests with different strains (i.e., 1000%, 3000%, and 5000% strains). As displayed in Figure S8a,b, the **PU-TASN-DTM/C** film owned a loosened amorphous microphase separated structure before stretching, which could be proven by the one-dimensional (1D) SAXS curve of the **PU-TASN-DTM/C** film, in which no apparent peak value could be observed. However, the hard domains of the **PU-TASN-DTM/C** film aggregated as the tensile strain extended, leading to the enlargement of the 1D SAXS curve with one dominant *q* value of ∼0.07 Å^–1^ owing to the average interdomain distance of 8.98 nm between oriented microphase-separated domains. With stretching up to 5000%, the 1D SAXS curve of the **PU-TASN-DTM/C** film decayed, since the partial segmentation of the hard domain structure appeared. Besides, the lower intensity might be related to the gradually cracked structure and a smaller thickness initiated from the rearrangement of the strain-encouraged structure in the stretched **PU-TASN-DTM/C** film. Moreover, the isotropic structure of the **PU-TASN-DTM/C** film in the unstretched state (at 0% strain), wherein the hard domains were unsystematically dispersed in PU matrices, could be confirmed through the two-dimensional (2D) SAXS patterns of the **PU-TASN-DTM/C** film in Figure S8a,b that afforded a uniform circular shape. The scattering circle in Figure S8b was regularly distorted and changed to two scattering spots oriented along the stretching direction with the increasing strain up to 5000%, suggesting that the hard domains constantly orientated along the stretching direction, and the orientation degree of the hard segments in the stretching direction was intensified, along with the aligned microstructure being caused under tensile forces.

Furthermore, as shown in Figure S8c,d, the 1D WAXS and 2D WAXS profiles of the **PU-TASN-DTM/C** film under various strains (i.e., 1000%, 3000%, and 5000% strains) could be employed to verify its structural transformations from static to tensile. At the strain of 5000%, as depicted in Figure S8c, the partial dissociation of hydrogen bonds might occur, accompanied by the possible enhancement of the molecular orientation and the probable reduction in the film thickness. Thus, the intensity of the scattering peak at 2θ ≈ 18 deg. (*q* ≈ 1.49 Å^–1^) for the **PU-TASN-DTM/C** film was weakened, proving the stacked structure with a period of ∼4.22 Å. Besides, the isotropic scattering halo of the 2D WAXS pattern in Figure S8d became centralized and transformed into two intense arcs (with 2θ ≈ 18° and a *d*-spacing value ∼4.22 Å) in the meridional direction with the augmenting strain, specifying that the favored orientation correlated with the periodic distance of PU backbones with stretching-generated hydrogen bonding in the tensile direction. These results declared that the incorporation of the movable [c2] daisy chain structures inhibited the shish-kebab orientation during stretching and could be a critical reason for mechanical performances of the elastomer to conserve the high toughness and stretchability.

### Shape Memory Performances

Interestingly, being one sort of SMP, PUs are able to recover their original shapes from deformed shapes in response to external stimulations. Therefore, excluding partial mechano-fluorescent restoration behaviors of **TASN** moieties in stretched PU films upon heating, the shape recoveries of PU films could also be examined since the enhanced hydrogen-bonds formed by the oriented elongations under tensile stresses could be degraded by thermal treatments. Thereafter, the mechanical–thermal cycles of the **PU-TASN-DTM/C** film were investigated and are shown in Figure S9 to study its shape memory performances. Whereas a blue fluorescent emission could be sensed in the force-free state of the **PU-TASN-DTM/C** film, an obvious yellow fluorescent emission became discernible in this PU film after stretching. Subsequently, the stretched **PU-TASN-DTM/C** film was frozen to protect its temporary shape and the yellow fluorescent emission of **DAAN** ^**•**^ species under tensile condition. Later, the temporary shape of the stretched **PU-TASN-DTM/C** film was able to almost revert to its undamaged shape (>90%) after immersion in hot water at approximately 70 °C. Furthermore, the shape recovery behaviors of the **PU-TASN-DTM/C** film could also be affirmed under several stretching–heating cycles as shown in Figure S7d, recommending the probable applications of resultant PU elastomers in manufacturing shape memory materials (see Figure S9 and Movies S6 and S7).

## Conclusion

Various force-active mechano-fluorescent PU elastomers were created by using step-growth polymerizations of distinct components, involving radical-type mechanofluorophoric **TASN** derivatives and AIE-active [c2] daisy chain rotaxanes in expanded/contracted conformations with different shuttling dimensions of axle- and macrocycle-exerted force modes (i.e., traditional **DTS/E** and **DTS/C** along with unconventional **DTM/C** and **DTM/E** daisy chain rotaxanes). Intriguingly, the insertion of quite low amounts (ca. 0.02% molar ratio of all monomer components) of daisy chain moieties (as artificial molecular muscle tougheners) into PU scaffolds could regulate mechanical properties of resultant mechano-fluorescent PU films efficiently. Hence, the optimized **PU-TASN-DTM/C** film possessing a daisy chain moiety **DTM/C** (by adding only 0.045% molar ratio of all diol monomers) demonstrated outstanding stretchability at a high strain rate of 20 mm/s (or 1200 mm/min). It also revealed a preeminent toughness up to 1363 MJ/m^3^ (i.e., almost 6.3 times greater than 215 MJ/m^3^ of original PU films without daisy chains) along with an impressive tensile strain of ∼5181% and a rough stress of 33.42 MPa. In contrast, the **PU-TASN-DTS/C** film showed an enhanced toughness of 836 MJ/m^3^, about 3.9 times higher than that of original PU films without daisy chains. Consequently, the unconventional **PU-TASN-DTM/C** film disclosed a preeminent toughness of 1363 MJ/m^3^ as the highest toughness record to the best of our knowledge, which was ca. 1.6 times tougher than 836 MJ/m^3^ of the traditional **PU-TASN-DTS/C** film due to their different shuttling dimensions of macrocycle- and axle-exerted force modes, respectively. Notably, the optimal **PU-TASN-DTM/C** film (weight = 20 mg) was able to lift a bottle weighing 3.0 kg, verifying its supreme toughness and stretchability with an exceptional loading weight ratio of 150,000 (i.e., *M*/*m* = 150,000). Additionally, the most appealing ratiometric fluorescent responses of blue-emitting TPE (λ_em_ = 470 nm) and yellow-emitting **DAAN** ^**•**^ (λ_em_ = 572 nm) moieties could be distinguished in the **PU-TASN-DTM/C** film due to the incorporation of contracted [c2] daisy chain rotaxane **DTM/C** into PU skeletons to facilitate the reversible dual fluorescence switching during mechanical stretching and relaxation processes of the PU film. Moreover, the ET efficiency of the stretched **PU-TASN-DTM/C** film could be determined to be 27.8% from the blue-emitting TPE donor to the mechano-fluorophoric yellow-emitting **DAAN** ^**•**^ acceptor, highlighting the distinctive ratiometric mechano-fluorescent switching behaviors of AIE-fluorophoric [c2] daisy chain-based PU films upon tensile forces. Furthermore, X-ray diffraction (XRD) measurements were conducted to investigate deformation behaviors of PU films under stretching to ascertain correlated morphological changes associated with oriented daisy chain rotaxane structures integrated within PU matrices. Besides, the designed AIE-active [c2] daisy chain-inserted PU films divulged dominant shape recovery performances (restoring over 90% of their pristine shape) and reversible ratiometric mechano-fluorescent emission variations upon heating (∼70 °C), enabling practical applications of functionalized PU elastomers to be exploited in numerous up-and-coming fields of advanced functional materials.

## Supplementary Material

















## Notes

Experimental details and additional data including ^1^H NMR, ^13^C NMR, HRMS, UV–vis absorption spectra, PL spectra, FTIR, TGA, and TRPL (PDF)
Synthetic [c2] daisy chain molecules’ reversible molecular shuttling of contracted **DTS/C** and expanded **DTS/E** conformations (MP4)
Synthetic [c2] daisy chain molecules’ reversible molecular shuttling of contracted **DTM/C** and expanded **DTM/E** conformations (MP4)
Creation of the mechano-sensitive elastomeric PUs containing **TASN** units by step-growth polymerizations (MP4)
A bottle weighing 3.0 kg being lifted by the prepared **PU-TASN-DTM/C** film (MP4)
Mechano-fluorescent emission switching of the **PU-TASN-DTM/C** film with dynamic strains up to 5000% (MP4)
Shape recovery behaviors of the **PU-TASN-DTM/C** film affirmed under several stretching–heating cycles under ambient light (MP4)
Shape recovery behaviors of the **PU-TASN-DTM/C** film affirmed under several stretching–heating cycles under the UV lamp (MP4)
