# Supplementary material for: Ratiometric Mechano-Fluorescent Elastomers Dually Promoted via Effective Force-Triggered Radicals and Preeminent Toughnesses/Stretchabilities by Unconventional Shuttling Dimensions of Tetraphenylethylene-Suspended [c2] Daisy Chain Rotaxanes
Source: ACS Mater Au. 2025 Nov 4;6(1):222–35. doi: 10.1021/acsmaterialsau.5c00182 (PMC12810041; doi:10.1021/acsmaterialsau.5c00182)
Supplement: Supplementary file 1 [file mg5c00182_si_001.pdf]

Supporting Information

Ratiometric Mechano-Fluorescent Elastomers Dually Promoted via Effective Force-Triggered Radicals and Preeminent Toughnesses/Stretchabilities by Unconventional Shuttling Dimension of Tetraphenylethylene-Suspended [c2] Daisy Chain Rotaxanes

Tu Thi Kim Cuc,[†] Ting-Chi Wu,[†] Pham Quoc Nhien,[§] Trang Manh Khang,[†] Shunmuga Nathan Shunmuga Nainar,[†] Bui Thi Buu Hue,[§] Wei-Tsung Chuang,[‡] Hsiu-Hui Chen,[¶] Michal Kohout,[‖] and Hong-Cheu Lin*,[†,#]

[†] Department of Materials Science and Engineering, National Yang Ming Chiao Tung University, Hsinchu 300093, Taiwan

[§] Department of Chemistry, College of Natural Sciences, Can Tho University, Can Tho City 94000, Viet Nam

[‡] National Synchrotron Radiation Research Center, Hsinchu 300092, Taiwan

[¶] Department of Molecular Science and Engineering, National Taipei University of Technology, Taipei 106335, Taiwan

[‖] Department of Organic Chemistry, University of Chemistry and Technology, Prague 16628, Czech Republic

[#] Center for Emergent Functional Matter Science, National Yang Ming Chiao Tung University, Hsinchu 300093, Taiwan

1. General Information

1.1. Materials

All required chemical reagents were purchased from commercial supplier sources of Alfa Aesar, Combi Blocks, Sigma-Aldrich, TCI, etc., and directly used without further purification. All solvents were dried/purified by the solvent purification system before using for reactions. All reactions were conducted under nitrogen gas and vacuum-line manipulations. Several chemicals and solvents with their abbreviations are listed as follows: 1,8-biazabicyclo[5.4.0] undec-7-ene (DBU), trifluoroacetic acid (TFA), *di-tert*-butyldicarbonate (Boc_2O), 4-dimethylaminopyridine (DMAP), 1-ethyl-3-(3-dimethylaminopropyl) carbodiimide hydrochloride (EDC), *p*-toluenesulfonyl chloride (TsCl), ethylenediamine tetraacetic acid disodium salt dihydrate ($Na_2EDTA.2H_2O$), tetra-ethylene glycol (TEG), hexamethylene diisocyanate (HDI), triethanolamine (TEA), dibutyltin dilaurate (DBTDL), dichloromethane (DCM), ethyl acetate (EtOAc), hexane (Hex), ethanol (EtOH), methanol (MeOH), tetrahydrofuran (THF), acetonitrile (MeCN), and dimethylformamide (DMF).

1.2. Instruments

Nuclear magnetic resonance (NMR) spectra were obtained from Varian Unity Inova 500 MHz and Varian VNMRS-600 NMR spectrometers operating at frequencies of 500 and 600 MHz at room temperature to confirm chemical structures of all synthetic compounds. High resolution mass spectra (HRMS) were recorded on a Bruker-Impact HD Mass Spectrometer by using an electrospray ionization (ESI) technique. The fluorescence emission and absorbance spectra of all acquired compounds were noticed by a Fluorescence Spectrophotometer (HITACHI F-7000) and an Ultraviolet-Visible Near-Infrared Spectrophotometer (Lambda 950, PerkinElmer), respectively. The fluorescence lifetime values of major samples were gained from the time-resolved photoluminescence (TRPL) profiles revealed on a PDL 200 Pulsed Diode Laser. The weights of all used materials were determined by a Mettler Toledo AG245 Analytical Balance. Fourier transform infrared spectroscopy (FTIR) PerkinElmer spectrum 100 was exploited to analyze target samples at room temperature by 16 scan times from 4000 to 400 cm^{-1} with a bandwidth of 4 cm^{-1}. The mechanical properties of polymer films were appraised by MTS Tytron 250 tensile system, and the thermal stability tests were studied by thermo-gravimetric analysis (TA instruments Q500, heating rate = 10 °C/min). X-ray diffraction experiments including small-angle X-ray scattering (SAXS) and wide-angle X-ray scattering (WAXS) measurements were achieved on TPS 13A and TLS 17A beamlines at the National Synchrotron Radiation Research Center (NSRRC) in Hsinchu, Taiwan.

1.3. Mechanical Testing

A MTS Tytron 250 tensile system was used to record all stress-strain curves. The polymer films with the dog-bone shaped specimens were fabricated. The middle sections of the dog-bone specimens were 5 mm in width and 10 mm in length. The thicknesses of the samples were identified by a micrometer, which varied from 0.6 mm to 0.7 mm. Before carrying out tensile strength tests, sample pieces were put on the jigs of the tension machine with an initial length of 1 mm, considered as the gap between two clips.

2. Experimental Section

2.1. Synthetic Routes

Scheme S1. Synthetic Routes of **TASN** and **TPE** Derivatives

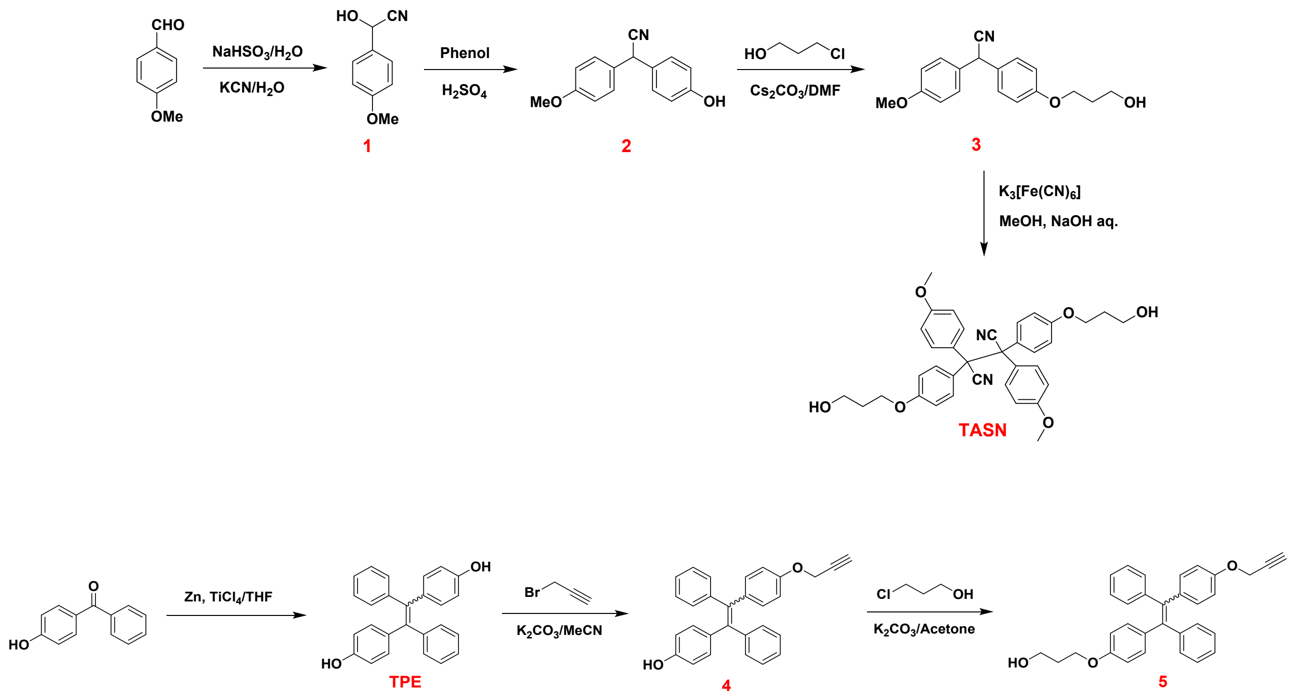

Scheme S2. Synthetic Routes of Alkyne **10** and DB24C8 Wheel-Decorating Secondary Ammonium Unit **15**

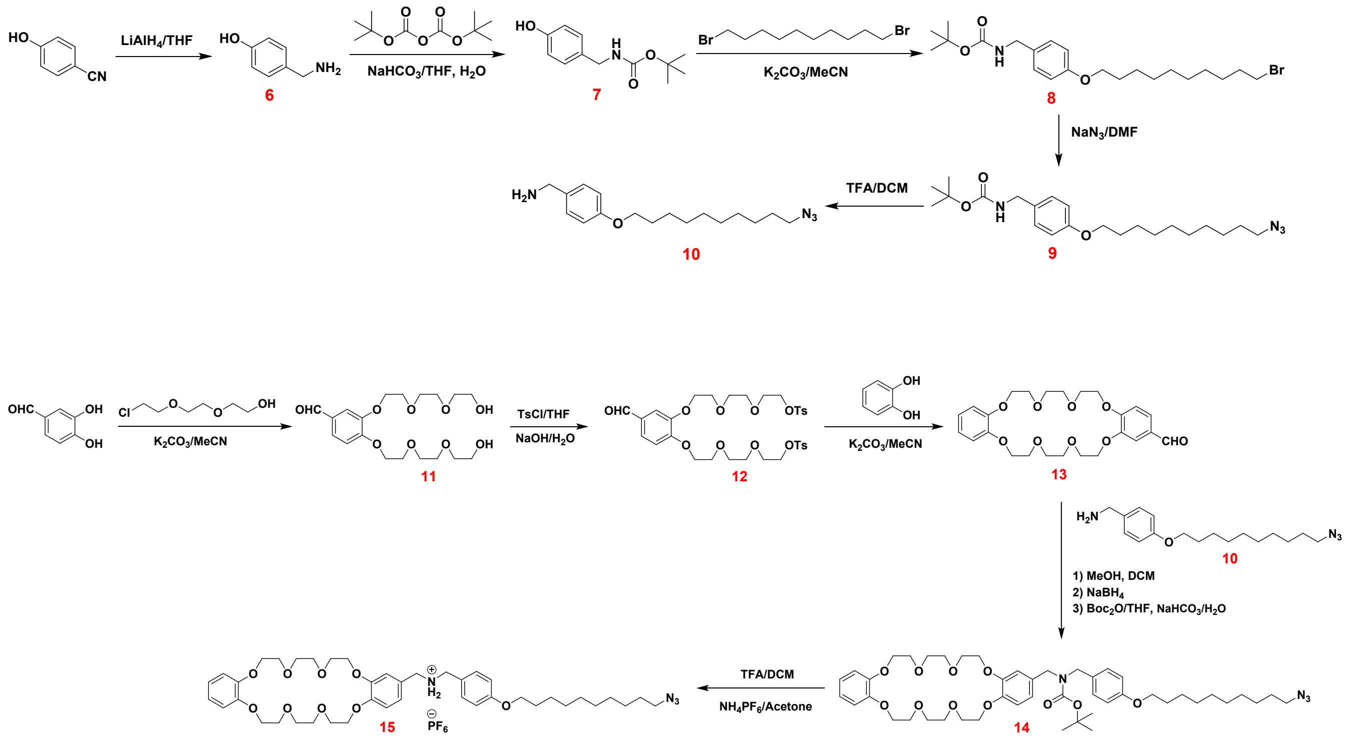

Scheme S3. Synthetic Routes of Daisy Chain Rotaxanes **DTS/E** and **DTS/C**

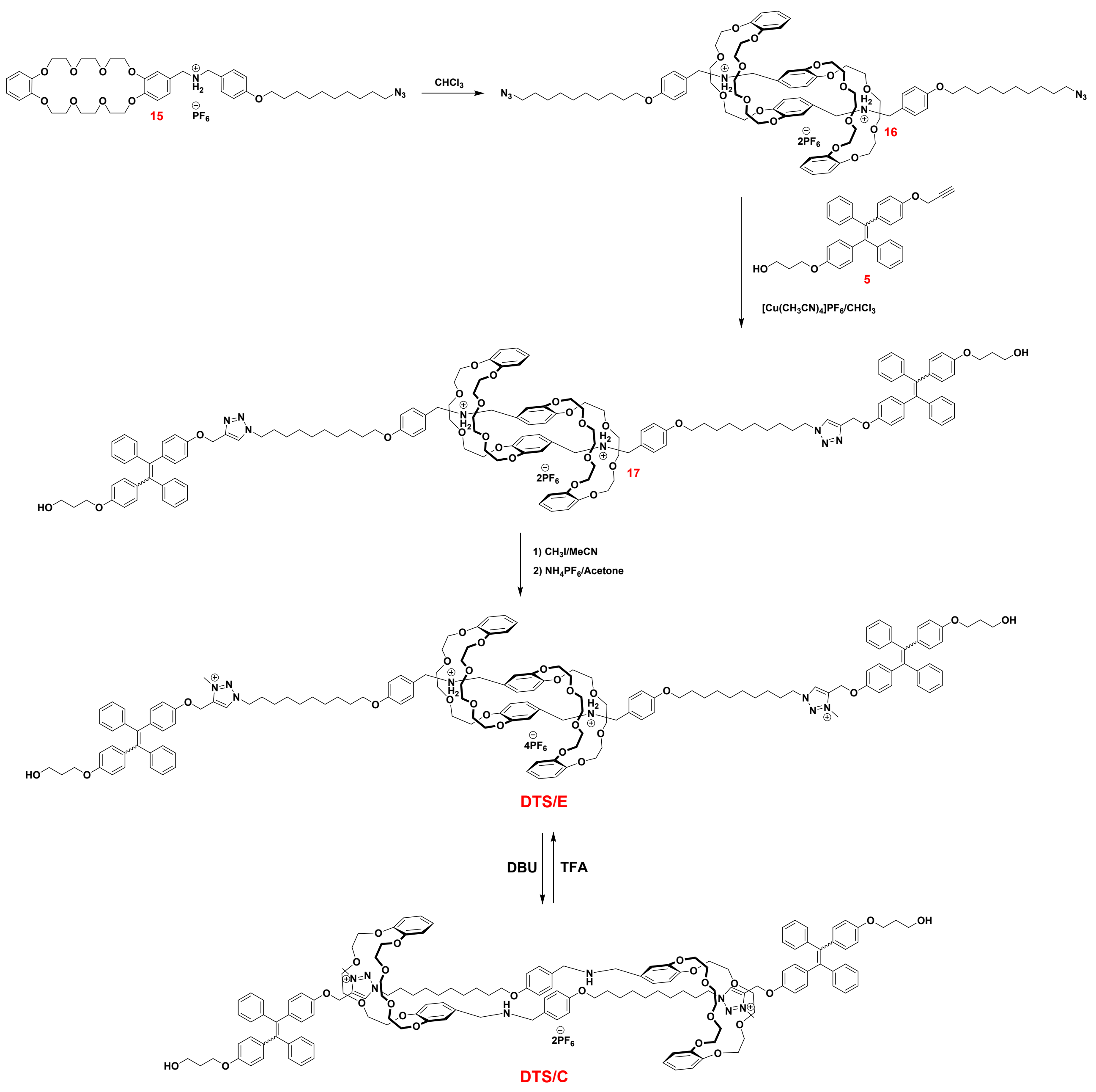

Scheme S4. Synthetic Routes of TPE Derivative **19** and Wheel-Decorating Secondary Ammonium Moiety **24**

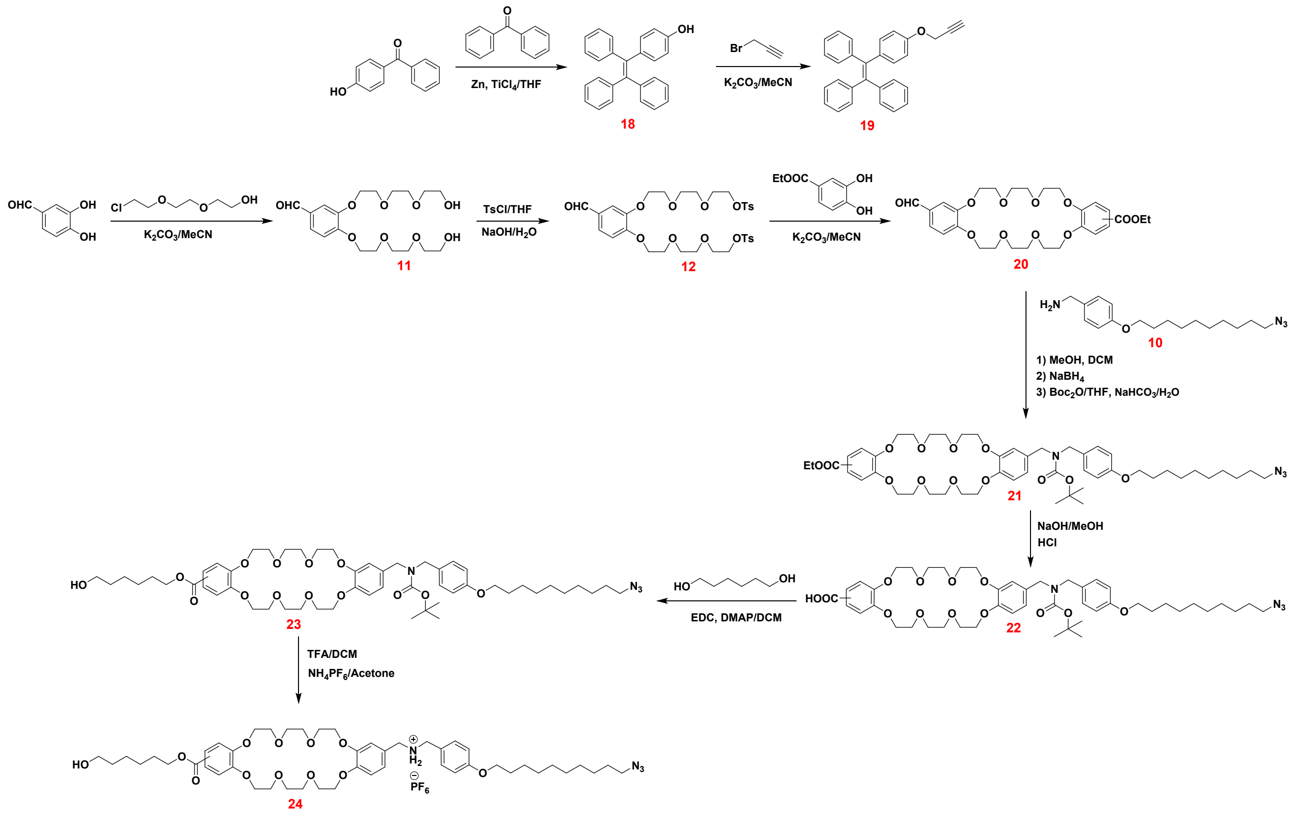

Scheme S5. Synthetic Routes of Daisy Chain Rotaxanes **DTM/C** and **DTM/E**

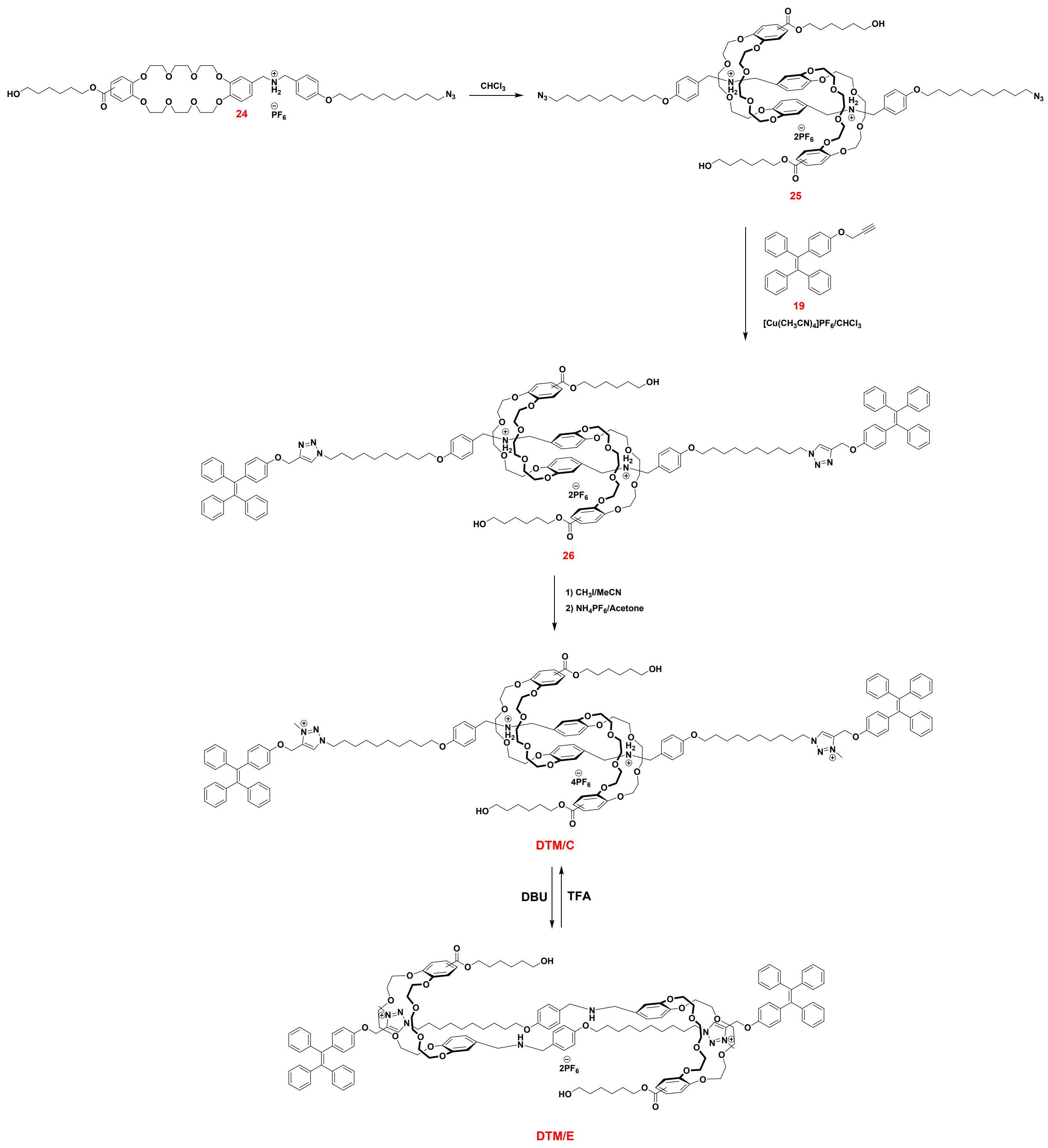

2.2. Synthetic Procedures and Characterizations of Intermediate Compounds and [c2] Daisy Chain Rotaxanes

Compounds **1-3** and **TASN** were synthesized according to the previous publication.[S1]

Compounds **TPE**, **4-17**, **20-22**, **DTS/E**, and **DTS/C** were synthesized according to our previous publication.[S2]

Compounds **18** and **19** were synthesized according to our previous publication.[S3]

Compounds **23** and **24** were synthesized according to our previous publication.[S4]

Compound TASN: ^{1}H NMR (500 MHz, $CDCl_3$, δ ppm): 7.17-7.14 (m, 8H), 6.77-6.73 (m, 8H), 4.09 (t, J = 5.0 Hz, 4H), 3.85 (t, J = 6.0 Hz, 4H), 3.78 (s, 6H), 2.05-2.00 (m, 4H). ^{13}C NMR (125 MHz, $CDCl_3$, δ ppm): 159.41, 158.63, 131.41, 131.38, 129.52, 129.31, 121.55, 113.85, 113.83, 113.36, 65.64, 60.34, 58.46, 55.37, 31.99. HRMS (ESI$^+$) [M+H]: calcd. for $C_{36}H_{37}N_2O_6$, 593.2652; found, 593.2646.

Synthesis of compound 26: Compound **24** (500 mg, 0.47 mmol) was dissolved in degassed $CHCl_3$ (30 mL) and stirred at room temperature for 3 h to yield compound **25**. Later, compound **19** (275 mg, 0.71 mmol) and $[Cu(CH_3CN)_4]PF_6$ (265 mg, 0.71 mmol) were directly added, and the reaction mixture was stirred at room temperature under nitrogen gas overnight. The resultant mixture was diluted with DCM (30 mL), stirred with an aqueous Na_2EDTA 0.1 M solution (50 mL), and washed with an aqueous solution of NH_4PF_6 (3×20 mL) and water. The organic layer was collected, dried over anhydrous Na_2SO_4, and concentrated under the reduced pressure. The crude product was purified by column chromatography with the solvent DCM:MeOH = 50:1 to acquire compound **26** as a yellow solid (480 mg, 70.8%). ^{1}H NMR (500 MHz, CD_3CN, δ ppm): 7.74 (s, 2H), 7.44-7.42 (m, 6H), 7.36-7.35 (m, 2H), 7.28-7.17 (m, 6H), 7.12-7.04 (m, 18H), 7.00-6.96 (m, 12H), 6.94-6.87 (m, 10H), 6.83-6.73 (m, 4H), 6.72-6.69 (m, 4H), 5.00 (s, 4H), 4.59-4.48 (m, 8H), 4.30-4.27 (m, 4H), 4.22-4.14 (m, 8H), 4.09-4.05 (m, 4H), 4.02-3.97 (m, 8H), 3.95-3.78 (m, 16H), 3.72-3.67 (m, 8H), 3.63-3.52 (m, 8H), 3.49-3.41 (m, 8H), 1.83-1.77 (m, 4H), 1.73-1.63 (m, 8H), 1.49-1.42 (m, 4H), 1.40-1.33 (m, 12H), 1.30-1.18 (m, 20H). ^{13}C NMR (125 MHz, CD_3CN, δ ppm): 165.89, 159.88, 159.77, 157.04, 151.78, 147.55, 146.69, 146.20, 145.99, 144.03, 143.97, 143.22, 140.66, 140.52, 136.50, 132.24, 131.01, 130.97, 130.83, 130.77, 130.61, 129.81, 127.84, 127.77, 126.53, 126.46, 126.44, 125.01, 123.72, 123.07, 122.84, 114.90, 114.01, 112.80, 112.10, 111.89, 111.36, 111.27, 71.88, 71.73, 71.46, 70.52, 70.38, 70.25, 69.77, 68.05, 67.74, 67.48, 64.77, 64.72, 61.55, 61.44, 51.97, 51.85, 51.59, 49.95, 32.53, 29.92, 29.15, 29.07, 29.01, 28.87, 28.62, 28.51, 26.08, 25.69, 25.38. HRMS (ESI$^+$) $[M-2PF_6^-]^{2+}$: calcd. for $C_{156}H_{190}N_8O_{26}^{2+}$, 1296.1907; found, 1296.1902.

Synthesis of compound DTM/C: Compound **26** (500 mg, 0.173 mmol) was dissolved in MeCN (10 mL), and then CH_3I (10 mL) was added into the solution of compound **26** in a sealed tube. Then, the reaction mixture was stirred at 45 °C for 2 days, and the mixture was cooled to room temperature. The excesses of MeCN and CH_3I were evaporated under the reduced pressure. The resultant solid was washed with Et_2O for several times, and then dispersed in acetone (30 mL). By the addition of an excessive amount of the saturated solution of NH_4PF_6, the achieved mixture was stirred at room temperature overnight. Following by the removal of the reaction solvent, the resulted residue was suspended in deionized water (50 mL), filtered and washed with water (3×50 mL). The precipitate was continuously washed with Et_2O and dried in a vacuum oven for 12 h to achieve the targeted [c2] daisy chain rotaxane **DTM/C** as a yellowish solid (430 mg, 77.6%). ^1H NMR (500 MHz, CD_3CN, δ ppm): 8.32 (s, 2H), 7.49-7.42 (m, 6H), 7.35-7.34 (m, 2H), 7.27-7.18 (m, 6H), 7.14-7.06 (m, 18H), 7.02-6.98 (m, 12H), 6.95-6.90 (m, 6H), 6.83-6.71 (m, 12H), 5.18 (s, 4H), 4.55-4.47 (m, 8H), 4.20-4.14 (m, 14H), 4.13-4.07 (m, 4H), 4.04-3.97 (m, 8H), 3.95-3.86 (m, 16H), 3.80-3.78 (m, 4H), 3.73-3.70 (m, 8H), 3.68-3.64 (m, 4H), 3.56-3.53 (m, 4H), 3.46-3.43 (m, 8H), 1.73-1.64 (m, 8H), 1.49-1.43 (m, 8H), 1.42-1.35 (m, 16H), 1.33-1.28 (m, 16H). ^{13}C NMR (125 MHz, CD_3CN, δ ppm): 165.82, 159.73, 155.73, 151.72, 147.48, 146.14, 145.92, 143.79, 143.72, 140.96, 140.24, 139.73, 137.88, 132.38, 130.87, 130.86, 130.83, 130.76, 130.70, 130.53, 130.01, 129.28, 128.64, 128.52, 127.82, 127.76, 127.73, 126.55, 126.49, 126.48, 123.92, 122.97, 122.80, 122.76, 114.96, 114.78, 114.74, 114.10, 71.83, 71.67, 70.46, 70.31, 70.19, 69.72, 69.59, 69.01, 68.06, 67.69, 67.42, 64.68, 64.63, 61.48, 58.17, 53.97, 51.92, 51.79, 38.41, 32.47, 29.19, 29.13, 29.04, 28.95, 28.86, 28.51, 28.45, 25.75, 25.63, 25.55, 25.31. HRMS (ESI$^+$) $[M-4PF_6^-]^{4+}$: calcd. for $C_{158}H_{196}N_8O_{26}^{4+}$, 655.6068; found, 655.6068.

Preparation of compound DTM/E: Compound **DTM/E** was prepared according to our previous publication with some modifications.[S2] The solution of compound **DTM/C** (500 mg, 0.156 mmol) in acetone (20 mL) was added with 1 M NaOH solution (3 mL) and stirred for 1 h at room temperature. After that, the solvent was removed under reduced pressure, and the obtained residue was dissolved in DCM (50 mL) and washed with de-ionized water (3×30 mL) as well as an aqueous solution of NH_4PF_6 (3×20 mL). Subsequently, the organic layer was dried over anhydrous Na_2SO_4, and the solvent was evaporated to give compound **DTM/E** as a yellowish solid (420 mg, 92.5%).

Preparation of PU films: All PU films were prepared according to the description in previous publications with some modifications,[S2,S5,S6] wherein the sample **PU-TASN-TPE** was a prototype. Compound **TASN** (29.6 mg, 0.05 mmol), TPE (1.82 mg, 0.005 mmol), tetraethylene glycol (TEG) (1068.3 mg, 5.5 mmol), and dibutyltin dilaurate (DBTDL) (1 drop) were dissolved in dry THF (8

mL) and refluxed under N_2 atmosphere for 15 min. Then, hexamethylene diisocyanate (HDI) (1009.2 mg, 6.0 mmol) was added and the resultant mixture was continuously reacted for 1 h. Later on, a solution of triethanolamine (TEA) (22.4 mg, 0.15 mmol) in dry THF (2 mL) was further added in the above mixture solution and nonstop reacted for further 15 min. Afterwards, the resultant solution was poured into a Teflon mold and dried in a vacuum oven at 70 °C for 1 day to yield a transparent straw-colored film.

Table S1. List of Abbreviations

**Abbreviations**	**Definitions**
PU	Polyurethane
TASN	Tetraarylsuccinonitrile
TPE	Tetraphenylethylene
DTS/C	The TPE-based daisy chain rotaxane with the axle-exerted force mode in the contracted form
DTS/E	The TPE-based daisy chain rotaxane with the axle-exerted force mode in the expanded form
DTM/C	The TPE-based daisy chain rotaxane with the macrocycle-exerted force mode in the contracted form
DTM/E	The TPE-based daisy chain rotaxane with the macrocycle-exerted force mode in the expanded form

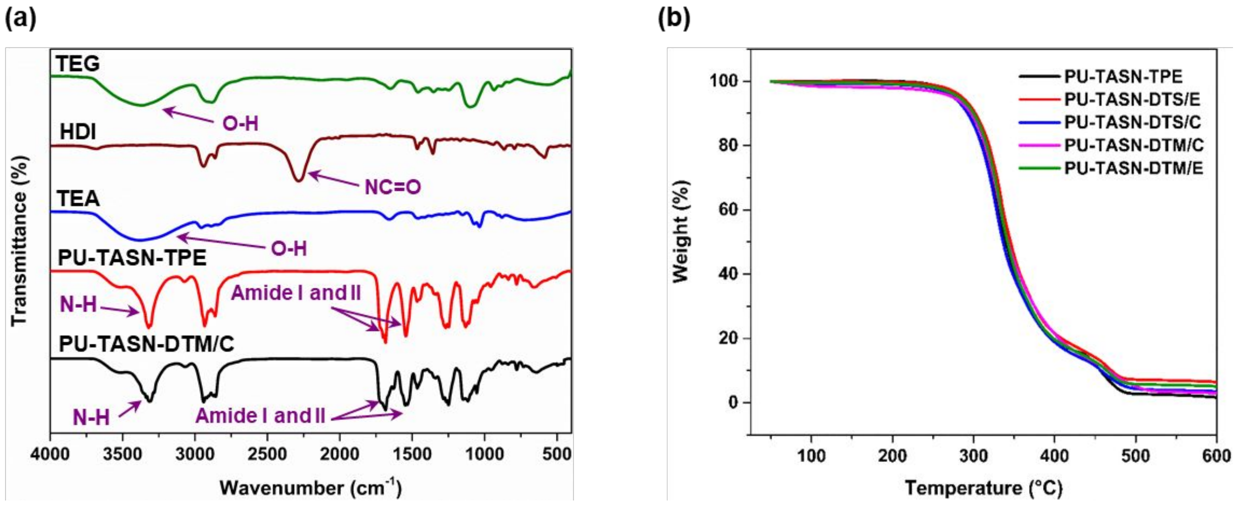

Figure S1. (a) FTIR spectra of TEG, HDI, TEA, **PU-TASN-TPE**, and **PU-TASN-DTM/C** films. (b) TGA curves of different PU films.

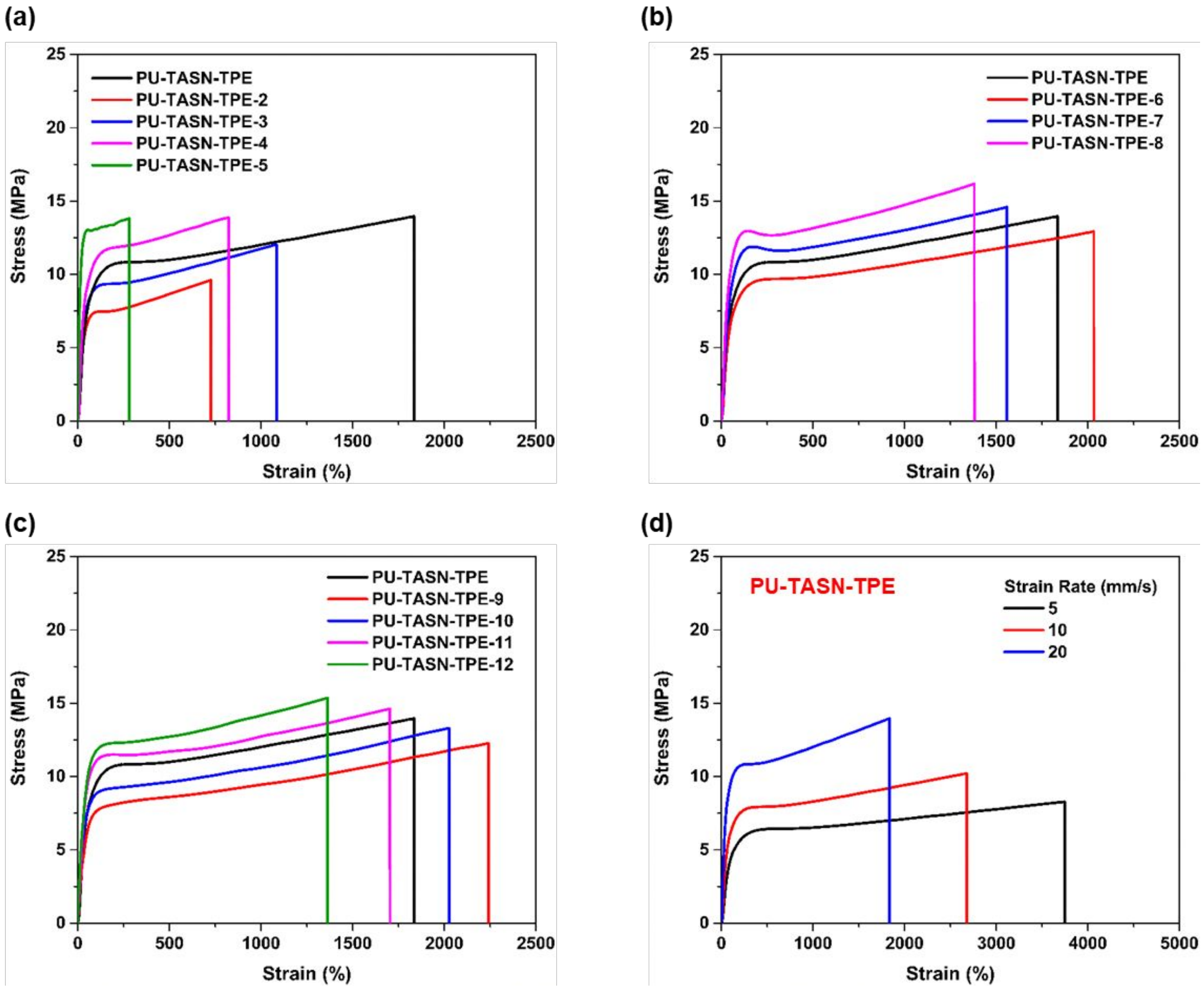

Figure S2. Stress-strain curves of **PU-TASN-TPE** films (the strain rate of 20 mm/s) with (a) different crosslinking densities, (b) distinct amounts of **TASN** mechano-fluorophore, and (c) various amounts of **TPE** unit under tensile loading. (d) Stress-strain curves of **PU-TASN-TPE** film with different strain rates (i.e., 5, 10, and 20 mm/s).

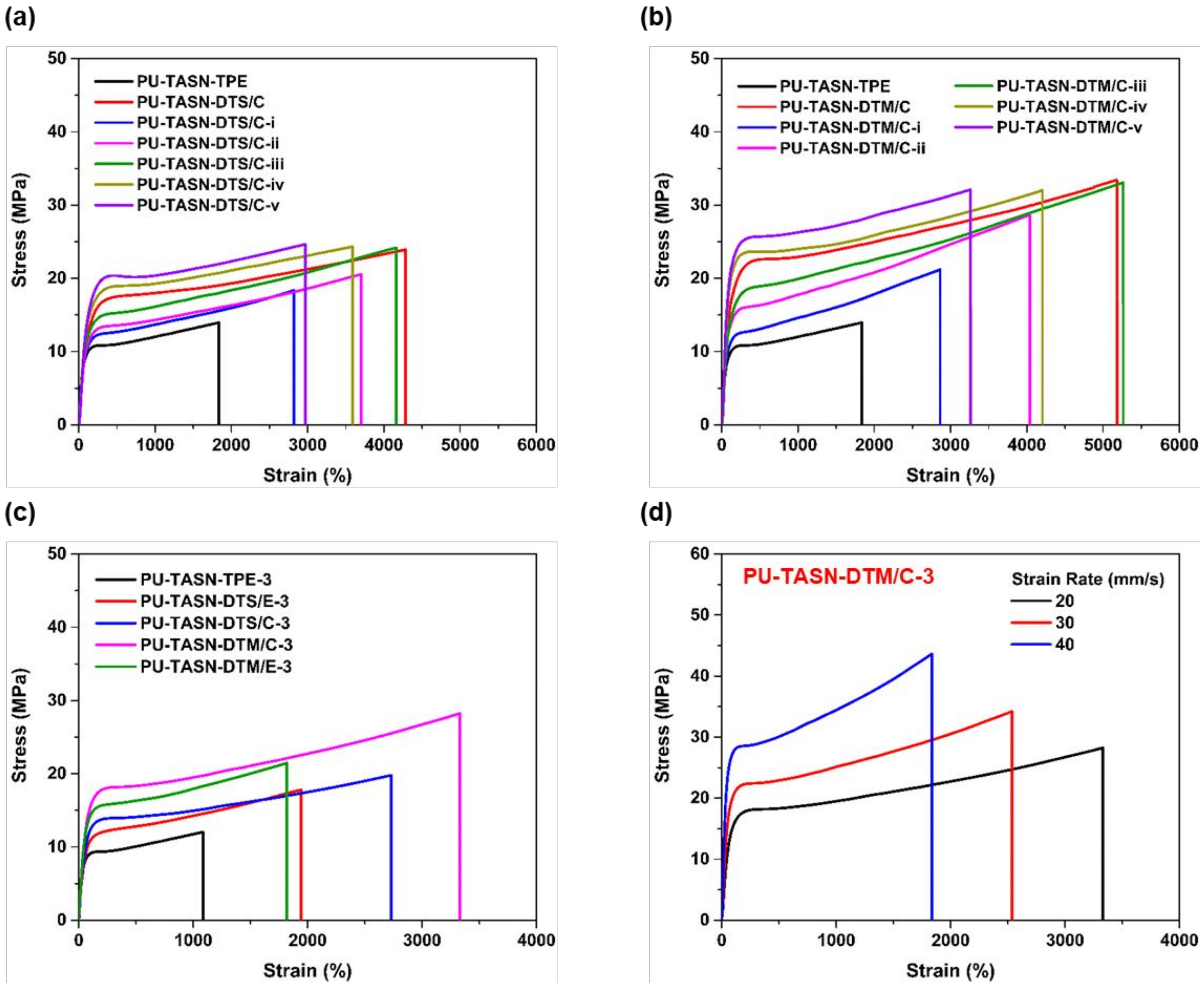

Figure S3. Stress-strain curves of (a) **PU-TASN-DTS/C** and (b) **PU-TASN-DTM/C** films (the strain rate of 20 mm/s) with different amounts of implanted daisy chain rotaxanes **DTS/C** and **DTM/C**. (c) Stress-strain curves of **PU-TASN-TPE-3**, **PU-TASN-DTS/E-3**, **PU-TASN-DTS/C-3**, **PU-TASN-DTM/C-3**, and **PU-TASN-DTM/E-3** films under tensile loading (the strain rate of 20 mm/s). (d) Stress-strain curves of **PU-TASN-DTM/C-3** film with various strain rates (i.e., 20, 30, and 40 mm/s).

Table S2. Mechanical Properties of PU (Including Optimized **PU-TASN-TPE**) Films Based on Various Molar Amounts of TEA, **TASN**, and **TPE**

PU Samples[a]	**TASN** (mmol)	**TPE** (mmol)	**TEG** (mmol)	**HDI** (mmol)	**TEA** (mmol)	Tensile Stress (MPa)	Tensile Strain (%)	Toughness (MJ/m^3)
	(eq.)	(eq.)	(eq.)	(eq.)	(eq.)			
**PU-TASN-TPE-1**	0.05	0.005	5.5	6.0	0	Non-Detectable (Powder)		
	1.0	0.1	110	120	0			
**PU-TASN-TPE-2**	0.05	0.005	5.5	6.0	0.05	9.61 ± 0.21	728 ± 12	58 ± 1
	1.0	0.1	110	120	1.0			
**PU-TASN-TPE-3**	0.05	0.005	5.5	6.0	0.10	12.04 ± 0.25	1085 ± 18	110 ± 2
	1.0	0.1	110	120	2.0			
**PU-TASN-TPE**[*]	0.05	0.005	5.5	6.0	0.15	13.96 ± 0.26	1835 ± 29	215 ± 3
	1.0	0.1	110	120	3.0			
**PU-TASN-TPE-4**	0.05	0.005	5.5	6.0	0.20	13.88 ± 0.29	824 ± 14	99 ± 2
	1.0	0.1	110	120	4.0			
**PU-TASN-TPE-5**	0.05	0.005	5.5	6.0	0.25	13.79 ± 0.24	282 ± 5	36 ± 1
	1.0	0.1	110	120	5.0			
**PU-TASN-TPE-6**	0.025	0.005	5.5	6.0	0.15	12.93 ± 0.30	2033 ± 32	217 ± 3
	0.5	0.1	110	120	3.0			
**PU-TASN-TPE-7**	0.075	0.005	5.5	6.0	0.15	14.59 ± 0.28	1560 ± 26	194 ± 3
	1.5	0.1	110	120	3.0			
**PU-TASN-TPE-8**	0.10	0.005	5.5	6.0	0.15	16.18 ± 0.34	1381 ± 24	189 ± 3
	2.0	0.1	110	120	3.0			
**PU-TASN-TPE-9**	0.05	0.00125	5.5	6.0	0.15	12.27 ± 0.29	2242 ± 40	217 ± 4
	1.0	0.025	110	120	3.0			
**PU-TASN-TPE-10**	0.05	0.0025	5.5	6.0	0.15	13.31 ± 0.26	2026 ± 36	216 ± 4
	1.0	0.05	110	120	3.0			
**PU-TASN-TPE-11**	0.05	0.0075	5.5	6.0	0.15	14.63 ± 0.32	1704 ± 28	211 ± 3
	1.0	0.15	110	120	3.0			
**PU-TASN-TPE-12**	0.05	0.01	5.5	6.0	0.15	15.36 ± 0.35	1363 ± 23	178 ± 3
	1.0	0.20	110	120	3.0			

[a] Mechanical tests of PU films were proceeded by using the strain rate of 20 mm/s (or 1200 mm/min).

[*] The optimized composition based on the molar amounts of TEA, **TASN**, and **TPE**.

Table S3. Mechanical Properties of PU (Including the Optimized **PU-TASN-DTS/C**) Films Based on Various Molar Amounts of **DTS/C**

PU Samples[a]	**TASN** (mmol)	**TPE** (mmol)	**TEG** (mmol)	**HDI** (mmol)	**TEA** (mmol)	**DTS/C** (mmol)	Tensile Stress (MPa)	Tensile Strain (%)	Toughness (MJ/m^3)
	(eq.)	(eq.)	(eq.)	(eq.)	(eq.)	(eq.)			
**PU-TASN-TPE**	0.05	0.005	5.5	6.0	0.15	0	13.96 ± 0.26	1835 ± 29	215 ± 3
	1.0	0.1	110	120	3.0	0			
**PU-TASN-DTS/C-i**	0.05	0	5.5	6.0	0.15	0.001	18.36 ± 0.36	2823 ± 50	410 ± 7
	1.0	0	110	120	3.0	0.02			
**PU-TASN-DTS/C-ii**	0.05	0	5.5	6.0	0.15	0.0015	20.56 ± 0.39	3702 ± 67	592 ± 11
	1.0	0	110	120	3.0	0.03			
**PU-TASN-DTS/C-iii**	0.05	0	5.5	6.0	0.15	0.002	24.18 ± 0.51	4163 ± 72	772 ± 13
	1.0	0	110	120	3.0	0.04			
**PU-TASN-DTS/C**[*]	0.05	0	5.5	6.0	0.15	0.0025	23.92 ± 0.45	4285 ± 73	836 ± 14
	1.0	0	110	120	3.0	0.05			
**PU-TASN-DTS/C-iv**	0.05	0	5.5	6.0	0.15	0.00375	24.33 ± 0.43	3589 ± 60	734 ± 12
	1.0	0	110	120	3.0	0.075			
**PU-TASN-DTS/C-v**	0.05	0	5.5	6.0	0.15	0.005	24.65 ± 0.50	2971 ± 49	624 ± 10
	1.0	0	110	120	3.0	0.10			

[a] Mechanical tests of PU films were proceeded by using the strain rate of 20 mm/s (or 1200 mm/min).

[*] The optimized composition of **PU-TASN-DTS/C** with the best mechanical property based on various molar amounts of **DTS/C**.

Table S4. Mechanical Properties of PU (Including Optimized **PU-TASN-DTM/C**) Films Based on Various Molar Amounts of **DTM/C**

PU Samples[a]	**TASN** (mmol) / (eq.)	**TPE** (mmol) / (eq.)	**TEG** (mmol) / (eq.)	**HDI** (mmol) / (eq.)	**TEA** (mmol) / (eq.)	**DTM/C** (mmol) / (eq.)	Tensile Stress (MPa)	Tensile Strain (%)	Toughness (MJ/m^3)
**PU-TASN-TPE**	0.05	0.005	5.5	6.0	0.15	0	13.96 ± 0.26	1835 ± 29	215 ± 3
	1.0	0.1	110	120	3.0	0			
**PU-TASN-DTM/C-i**	0.05	0	5.5	6.0	0.15	0.001	21.19 ± 0.42	2863 ± 48	456 ± 8
	1.0	0	110	120	3.0	0.02			
**PU-TASN-DTM/C-ii**	0.05	0	5.5	6.0	0.15	0.0015	28.66 ± 0.51	4038 ± 69	852 ± 15
	1.0	0	110	120	3.0	0.03			
**PU-TASN-DTM/C-iii**	0.05	0	5.5	6.0	0.15	0.002	33.07 ± 0.69	5262 ± 89	1287 ± 22
	1.0	0	110	120	3.0	0.04			
**PU-TASN-DTM/C**[*]	0.05	0	5.5	6.0	0.15	0.0025	33.42 ± 0.63	5181 ± 84	1363 ± 22
	1.0	0	110	120	3.0	0.05			
**PU-TASN-DTM/C-iv**	0.05	0	5.5	6.0	0.15	0.00375	32.01 ± 0.61	4204 ± 71	1103 ± 19
	1.0	0	110	120	3.0	0.075			
**PU-TASN-DTM/C-v**	0.05	0	5.5	6.0	0.15	0.005	32.13 ± 0.59	3259 ± 56	893 ± 15
	1.0	0	110	120	3.0	0.10			

[a] Mechanical tests of PU films were proceeded by using the strain rate of 20 mm/s (or 1200 mm/min).

[*] The optimized composition of **PU-TASN-DTM/C** with the best mechanical property based on various molar amounts of **DTM/C**.

Table S5. Mechanical Properties of **PU-TASN-DTS/E** and **PU-TASN-DTM/E** Films

PU Samples[a]	**TASN** (mmol) (eq.)	**TPE** (mmol) (eq.)	**TEG** (mmol) (eq.)	**HDI** (mmol) (eq.)	**TEA** (mmol) (eq.)	**DTS/E** (mmol) (eq.)	**DTM/E** (mmol) (eq.)	Tensile Stress (MPa)	Tensile Strain (%)	Toughness (MJ/m^3)
**PU-TASN-TPE**	0.05	0.005	5.5	6.0	0.15	0	0	13.96 ± 0.26	1835 ± 29	215 ± 3
	1.0	0.1	110	120	3.0	0	0			
**PU-TASN-DTS/E**	0.05	0	5.5	6.0	0.15	0.0025	0	22.03 ± 0.40	2945 ± 53	520 ± 9
	1.0	0	110	120	3.0	0.05	0			
**PU-TASN-DTM/E**	0.05	0	5.5	6.0	0.15	0	0.0025	25.65 ± 0.48	2782 ± 50	586 ± 11
	1.0	0	110	120	3.0	0	0.05			

[a] Mechanical tests of PU films were proceeded by using the strain rate of 20 mm/s (or 1200 mm/min).

Table S6. Mechanical Properties of **PU-TASN-DTM/C** Film and Most Recently Reported PU-Based Elastomers

Reference Number	Polymeric Elastomer	Tensile Stress (MPa)	Tensile Strain (%)	Toughness (MJ/m^3)	Strain Rate (mm/min)
S7	Poly(urethane-urea)	63.3	1511	430.9	50
S8	Poly(urethane-urea)	76.4	839	308.6	N/A[a]
S9	Polyurethane	15.1	910	N/A[a]	N/A[a]
S10	Poly(urethane-urea)	75.8	711	171.4	50
S11	Polyurethane	57.0	2260	460.0	100
S12	Polyurethane	24.5	1485	156.4	50
S13	Polyurethane	92.2	1932	480.2	50
S14	Polyurethane	78.3	1273	505.7	100
S15	Polyurethane	46.6	1737	337.2	100
S16	Poly(urethane-urea)	117.5	588	273.8	50
**This work**	**Polyurethane**	**33.4**	**5181**	**1363**	**1200**[b]

[a] N/A: Not available.

[b] Due to the unbreakable samples under tensile tests at lower strain rates of 50-100 mm/min normally reported in the listed references, the promoted values better than this record-high toughness of 1363 MJ/m^3 should be accessible at lower strain rates than 1200 mm/min (or 20 mm/s) utilized in our study.

Table S7. Mechanical Properties of PU Films Consisting of Different Types of [c2] Daisy Chain Rotaxane Molecules

PU Samples[a]	Tensile Stress (MPa)	Tensile Strain (%)	Toughness (MJ/m^3)
**PU-TASN-TPE-3**	12.04 ± 0.25	1085 ± 18	110 ± 2
**PU-TASN-DTS/E-3**	17.79 ± 0.30	1946 ± 33	274 ± 5
**PU-TASN-DTS/C-3**	19.78 ± 0.40	2731 ± 48	433 ± 8
**PU-TASN-DTM/C-3**	28.24 ± 0.54	3333 ± 58	723 ± 12
**PU-TASN-DTM/E-3**	21.46 ± 0.37	1818 ± 31	319 ± 5

[a] Mechanical tests of PU films were proceeded by using the strain rate of 20 mm/s (or 1200 mm/min).

Table S8. Mechanical Properties of **PU-TASN-TPE** Film with Different Strain Rates

Strain Rate (mm/s)	Tensile Stress (MPa)	Tensile Strain (%)	Toughness (MJ/m^3)
5	8.29 ± 0.15	3748 ± 67	261 ± 5
10	10.22 ± 0.18	2680 ± 50	230 ± 4
20	13.96 ± 0.26	1835 ± 29	215 ± 3

Table S9. Mechanical Properties of **PU-TASN-DTM/C-3** Film with Different Strain Rates

Strain Rate (mm/s)	Tensile Stress (MPa)	Tensile Strain (%)	Toughness (MJ/m^3)
20	28.24 ± 0.54	3333 ± 58	723 ± 12
30	34.22 ± 0.58	2538 ± 44	673 ± 12
40	43.65 ± 0.83	1836 ± 33	621 ± 11

Table S10. Load-Bearing Capacity of **PU-TASN-DTM/C** Film and Most Recently Reported PU-Based Elastomers

Reference Number	Polymeric Elastomer	Loaded Weight (kg) (M)	Polymer Weight (g) (m)	Load-Bearing Capacity (Ratio) (M/m)
S7	Poly(urethane-urea)	3.0	0.12	25,000
S8	Poly(urethane-urea)	5.5	0.29	~19,000
S9	Polyurethane	1.5	0.208	~7,200
S10	Poly(urethane-urea)	26.0	1.5	~17,000
S11	Polyurethane	5.0	0.05	100,000
S12	Polyurethane	5.0	0.04	125,000
S13	Polyurethane	10.0	0.1	100,000
S14	Polyurethane	7.3	0.162	~45,000
S15	Polyurethane	7.3	0.24	~30,000
S16	Poly(urethane-urea)	5.0	0.04	125,000
**This work**	**Polyurethane**	**3.0**	**0.02**	**150,000**

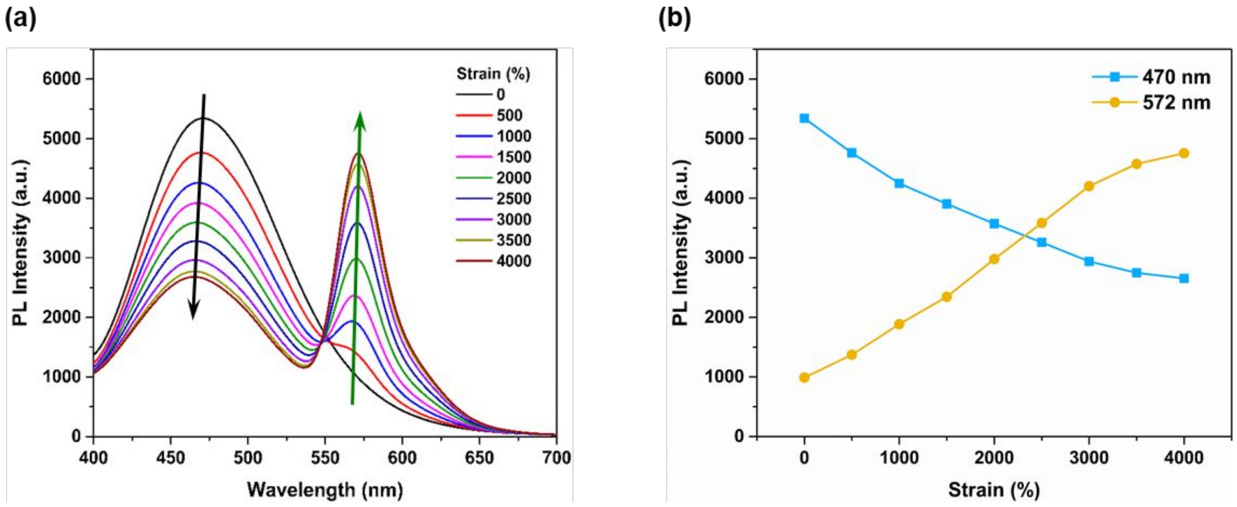

Figure S4. (a) PL spectra and (b) relative PL intensities of blue-emissive TPE (λ_{em} = 470 nm) and yellow-emissive **DAAN·** (λ_{em} = 572 nm) for **PU-TASN-DTS/C** film with different strains (λ_{ex} = 365 nm).

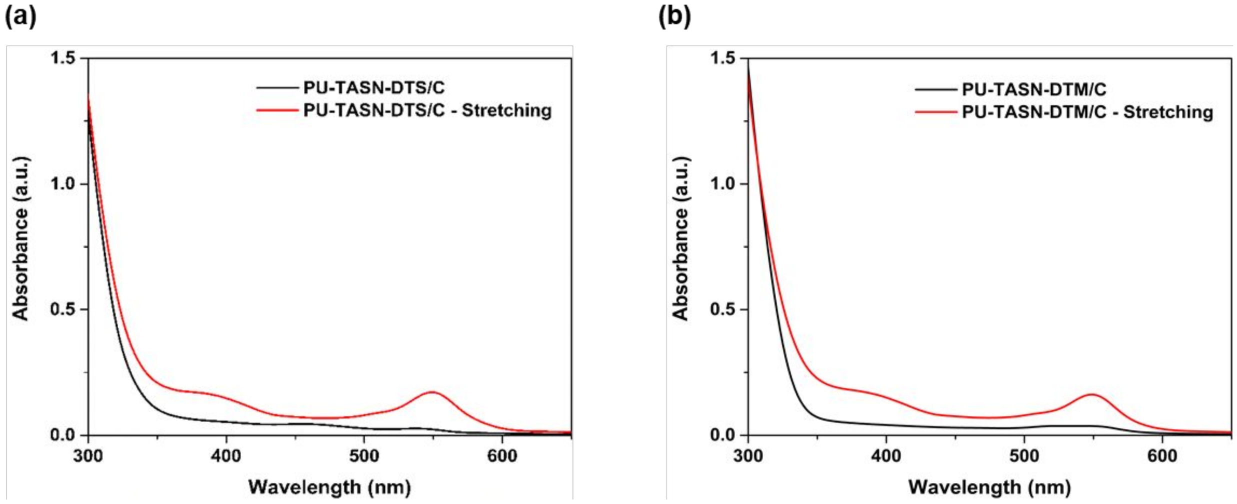

Figure S5. UV-vis spectra of (a) **PU-TASN-DTS/C** and (b) **PU-TASN-DTM/C** films before and after stretching.

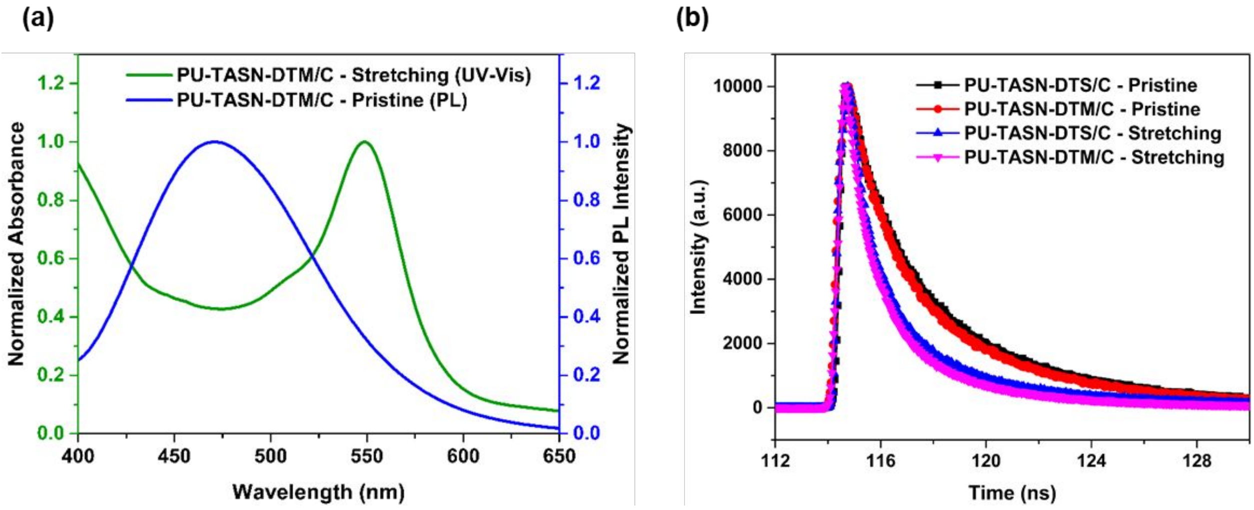

Figure S6. (a) Spectral overlap between the emission spectrum of pristine **PU-TASN-DTM/C** film and absorption spectrum of stretched **PU-TASN-DTM/C** film (λ_{ex} = 365 nm). (b) TRPL profiles of **PU-TASN-DTS/C** and **PU-TASN-DTM/C** films before and after stretching (λ_{ex} = 375 nm, λ_{em} = 470 nm).

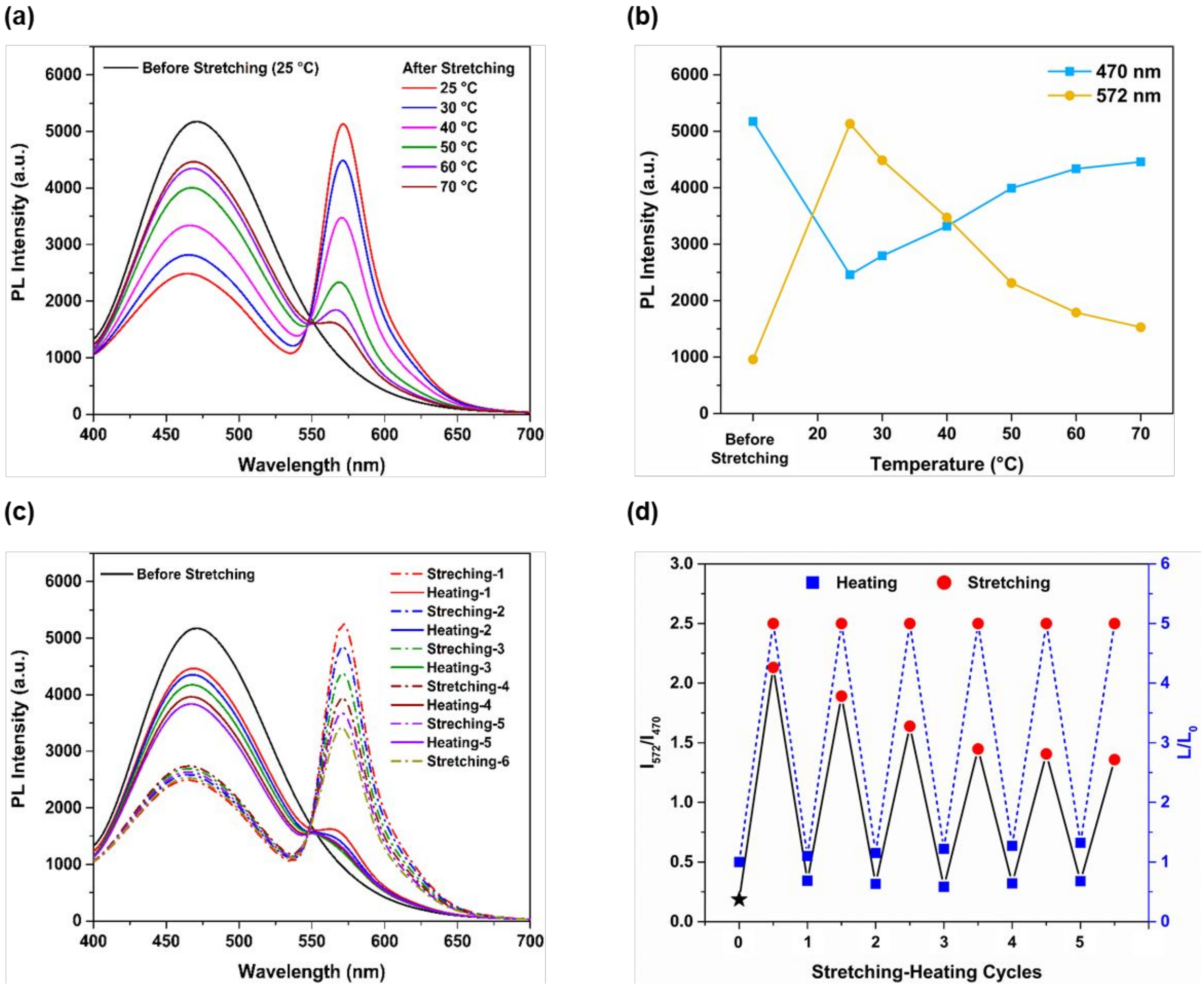

Figure S7. (a) PL spectra and (b) relative PL intensities of blue-emissive TPE (λ_{em} = 470 nm) and yellow-emissive **DAAN·** units (λ_{em} = 572 nm) for **PU-TASN-DTM/C** film at different temperatures after stretching. (c) PL spectra and (d) PL intensity ratios of two emission bands at 470 and 572 nm along with the length ratios for **PU-TASN-DTM/C** film by 5 cycles of stretching and thermal treatments (heating at 70 °C) (λ_{ex} = 365 nm).

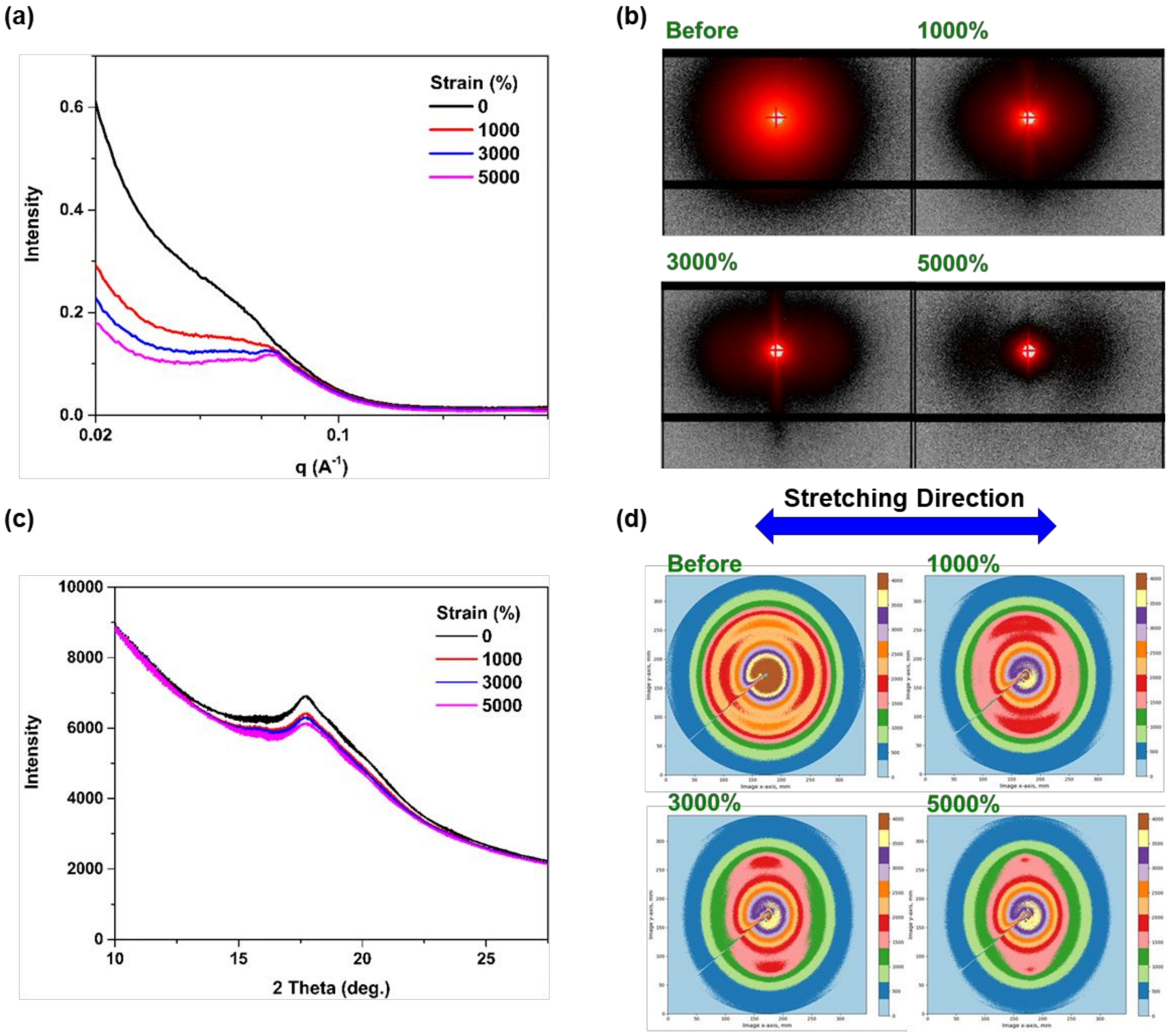

Figure S8. (a) 1D SAXS profiles, (b) 2D SAXS images, (c) 1D WAXS profiles, and (d) 2D WAXS images of **PU-TASN-DTM/C** film before and after stretching of 1000%, 3000%, and 5000% strains.

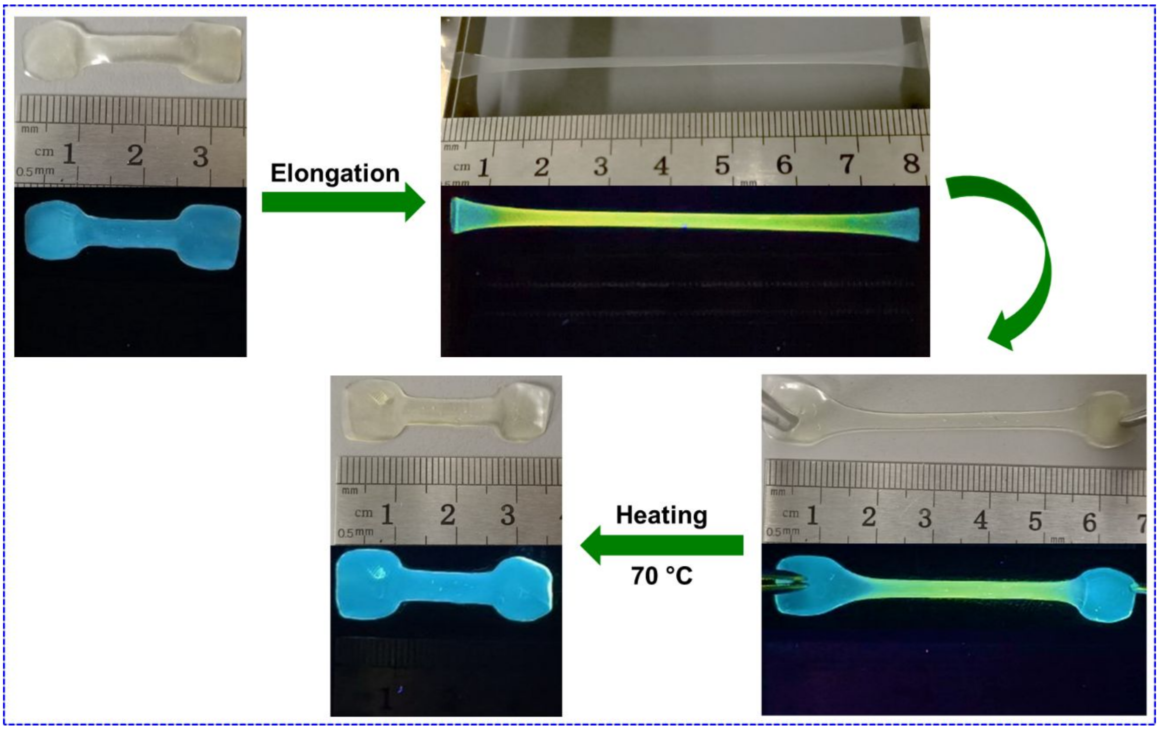

Figure S9. Demonstration of **PU-TASN-DTM/C** film with a responsive shape memory effect and emission color changes upon stretching and heating (ca. 70 °C). The top images were taken under the ambient light and bottom images were taken under the UV lamp (λ = 365 nm).

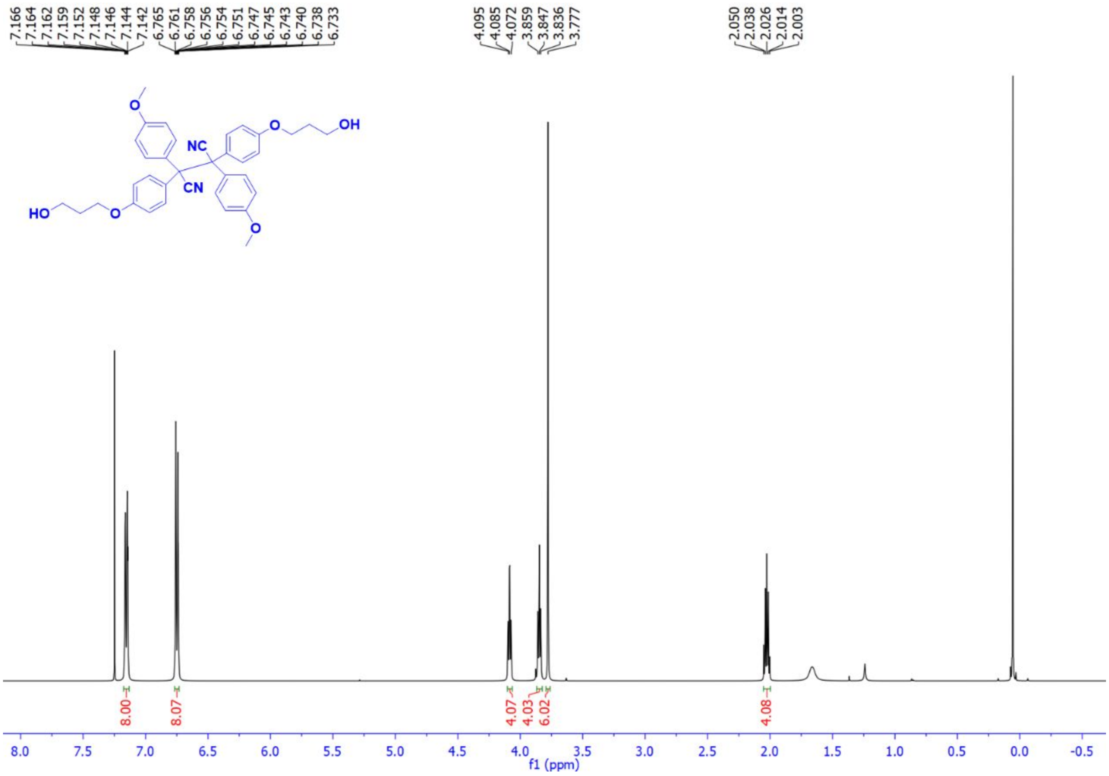

Figure S10. ^{1}H NMR spectrum (500 MHz, $CDCl_3$) of compound **TASN**.

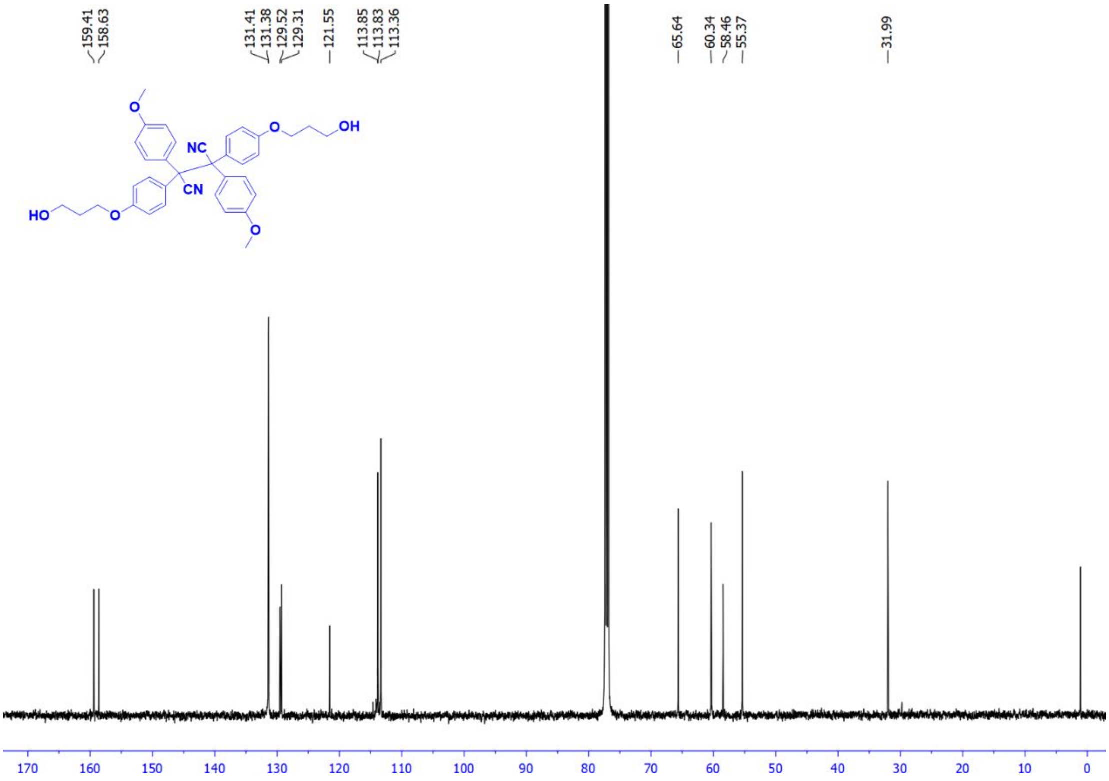

Figure S11. ^{13}C NMR spectrum (125 MHz, $CDCl_3$) of compound **TASN**.

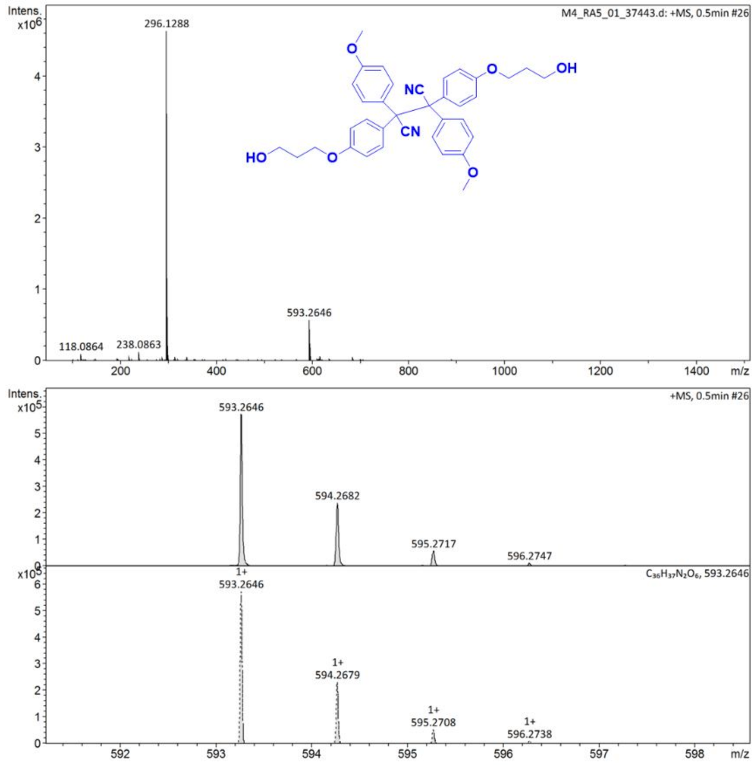

Figure S12. HRMS-ESI spectra of compound **TASN**.

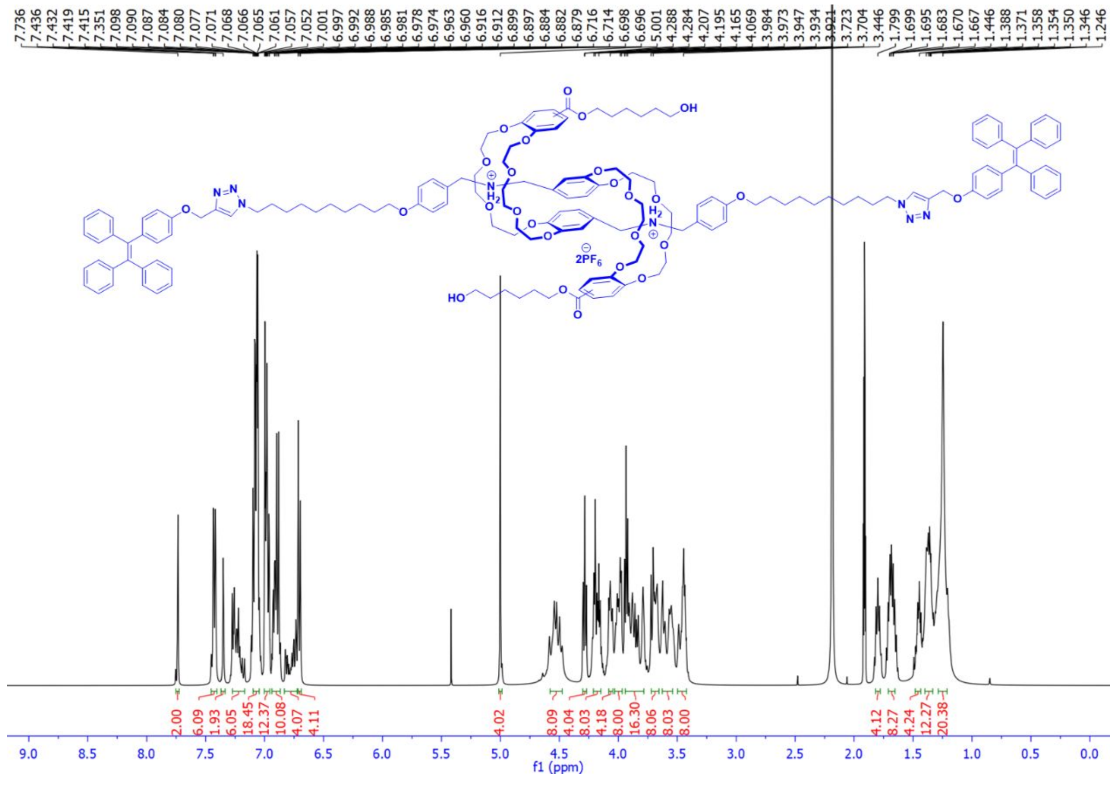

Figure S13. ^{1}H NMR spectrum (500 MHz, CD_3CN) of compound **26**.

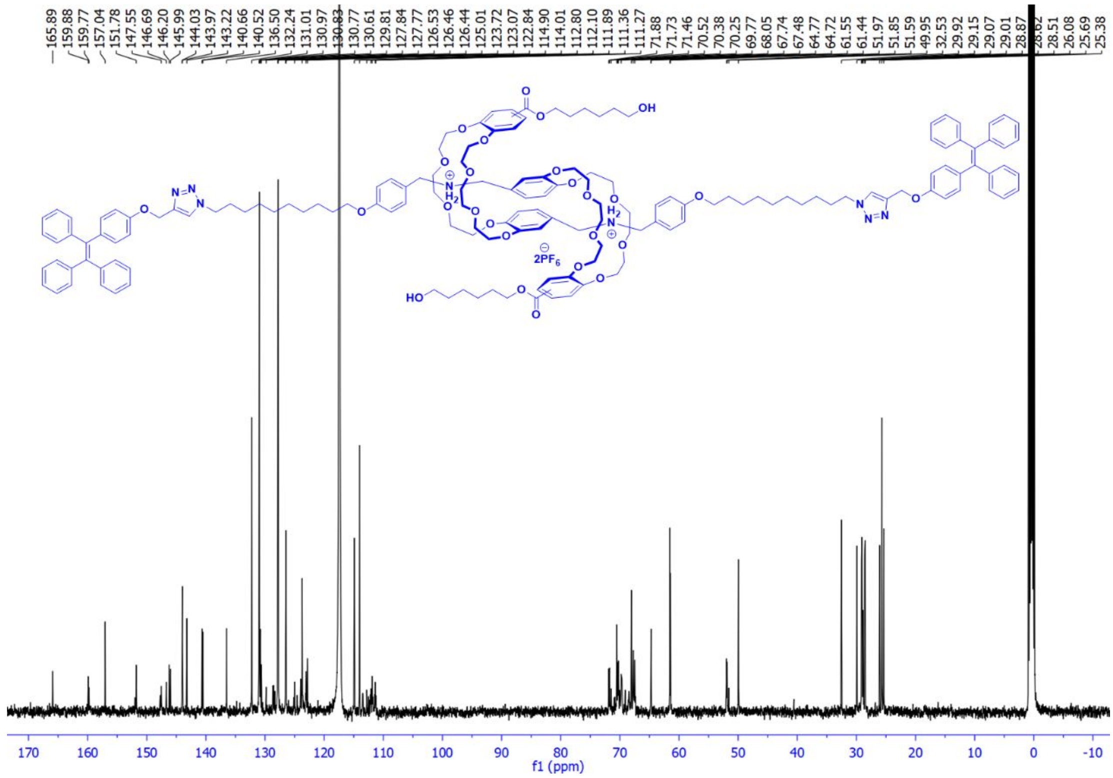

Figure S14. ^{13}C NMR spectrum (125 MHz, CD_3CN) of compound **26**.

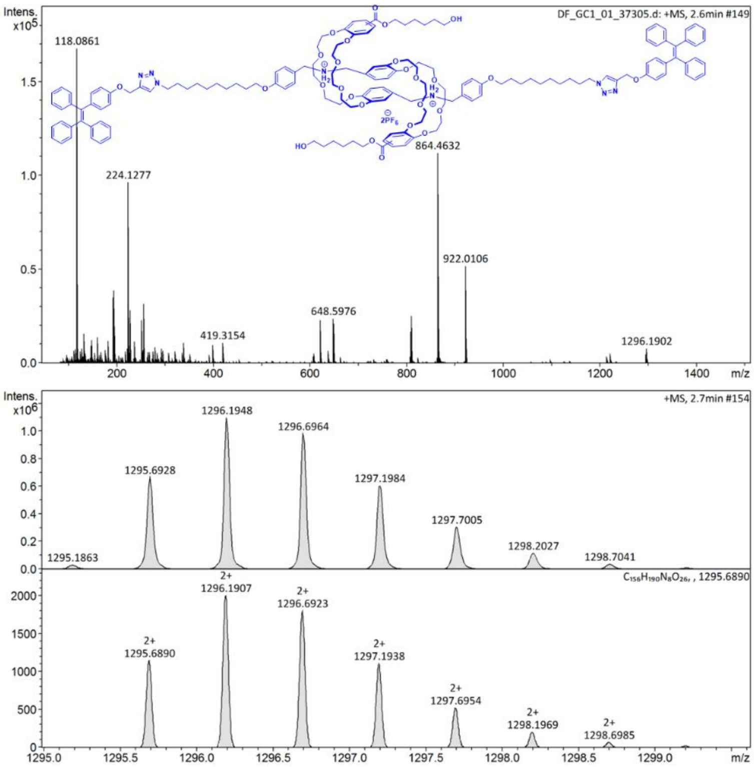

Figure S15. HRMS-ESI spectra of compound **26**.

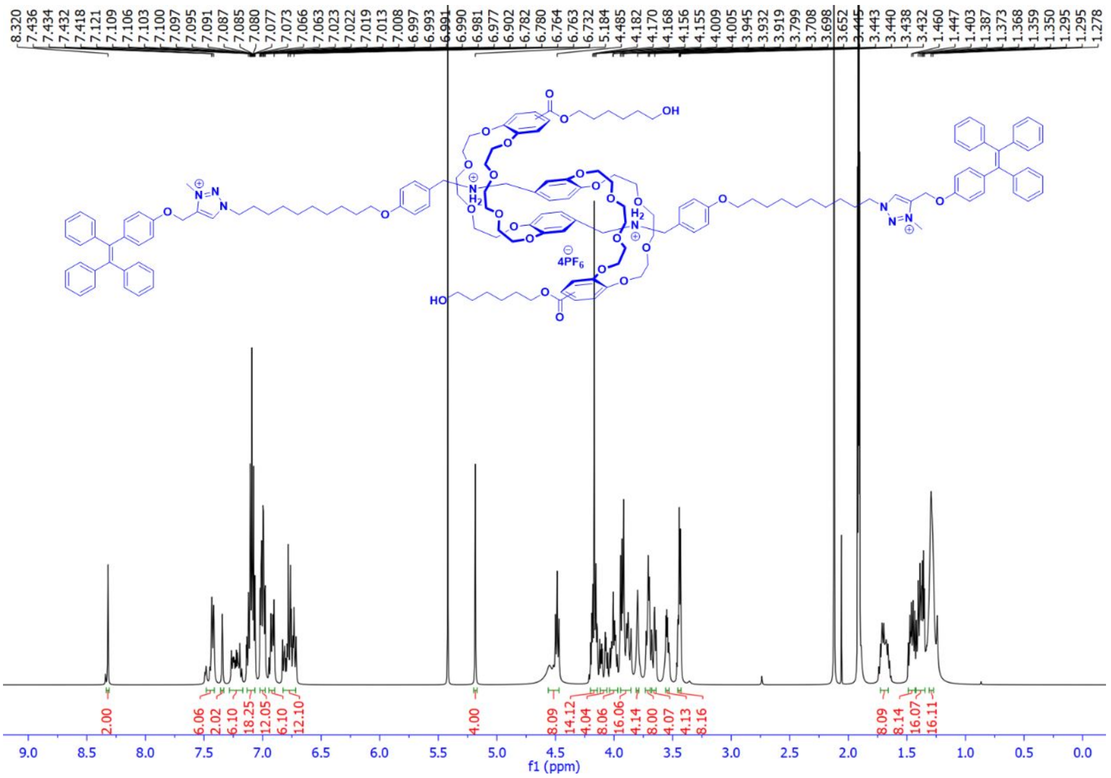

Figure S16. ^{1}H NMR spectrum (500 MHz, CD_3CN) of compound **DTM/C**.

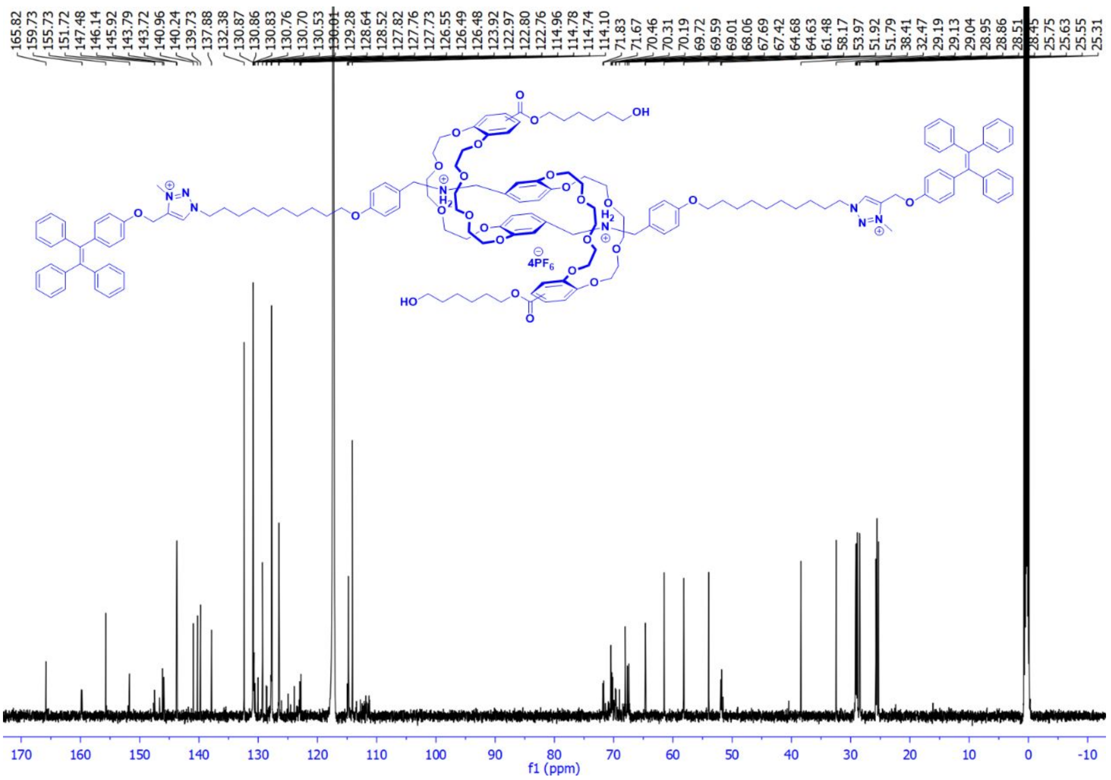

Figure S17. ^{13}C NMR spectrum (125 MHz, CD_3CN) of compound **DTM/C**.

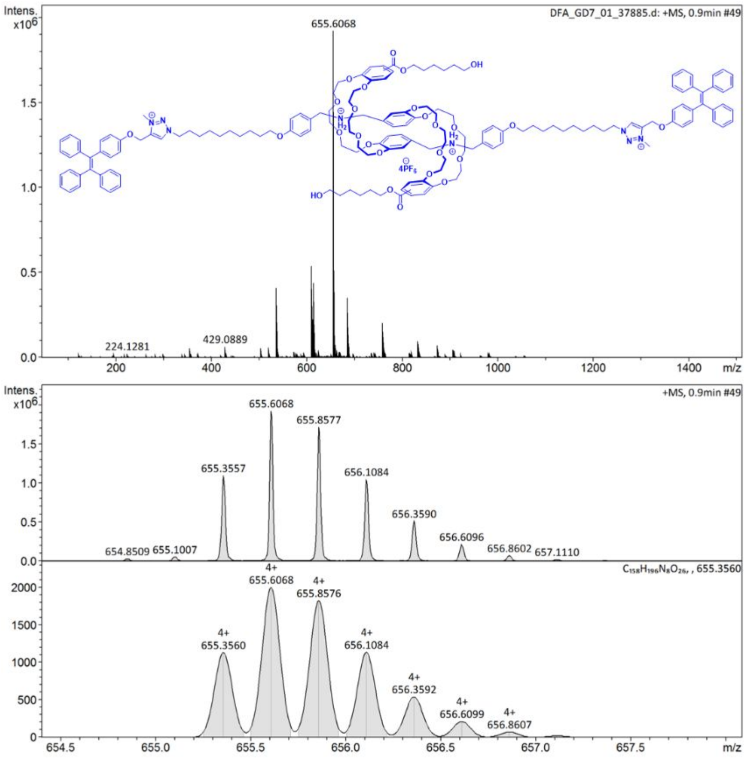

Figure S18. HRMS-ESI spectra of compound **DTM/C**.

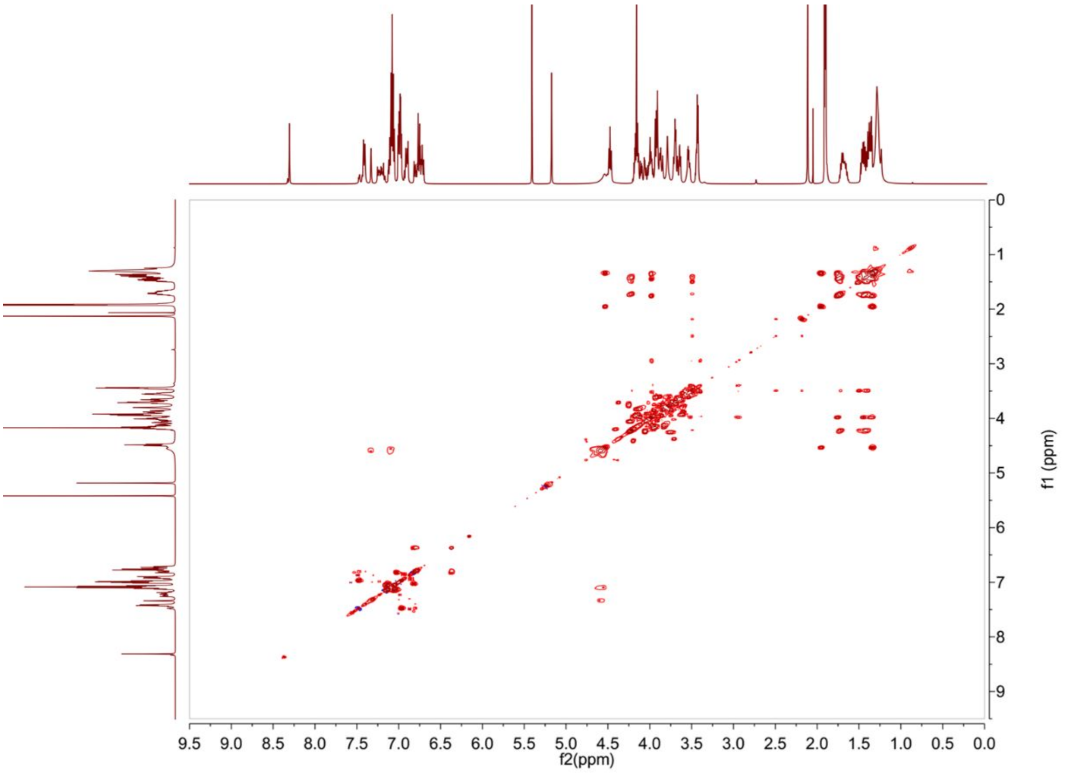

Figure S19. TOCSY NMR spectrum (600 MHz, CD_3CN) of compound **DTM/C**.

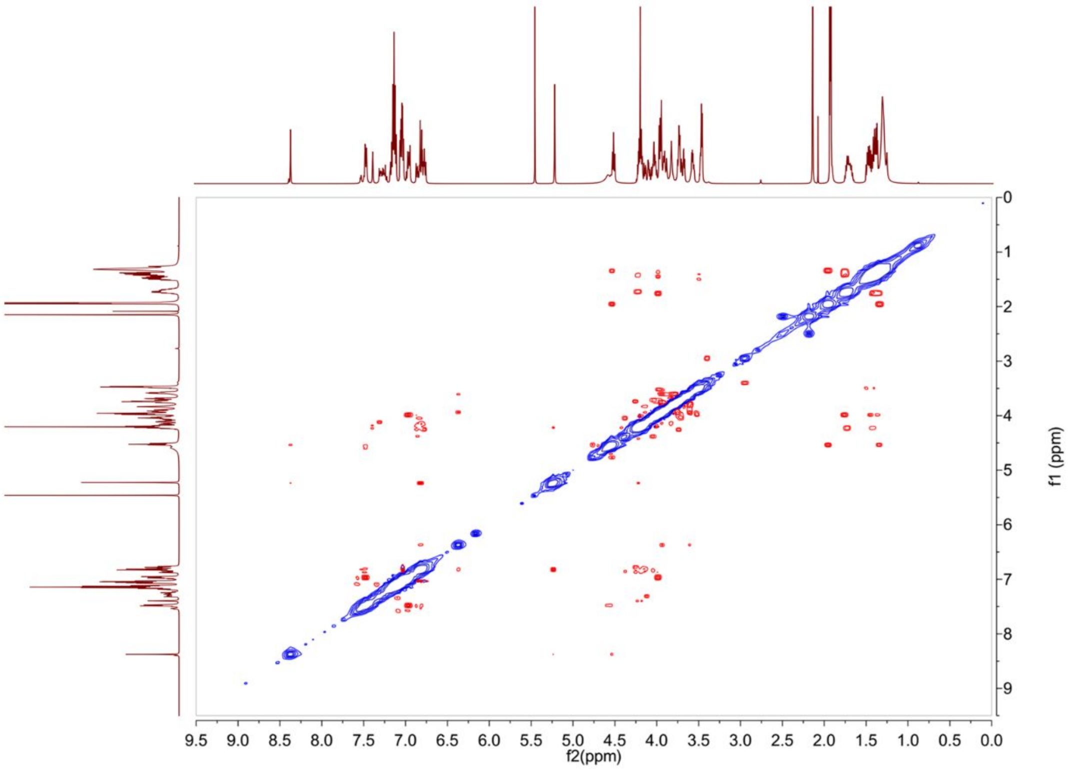

Figure S20. ROESY NMR spectrum (600 MHz, CD_3CN) of compound **DTM/C**.

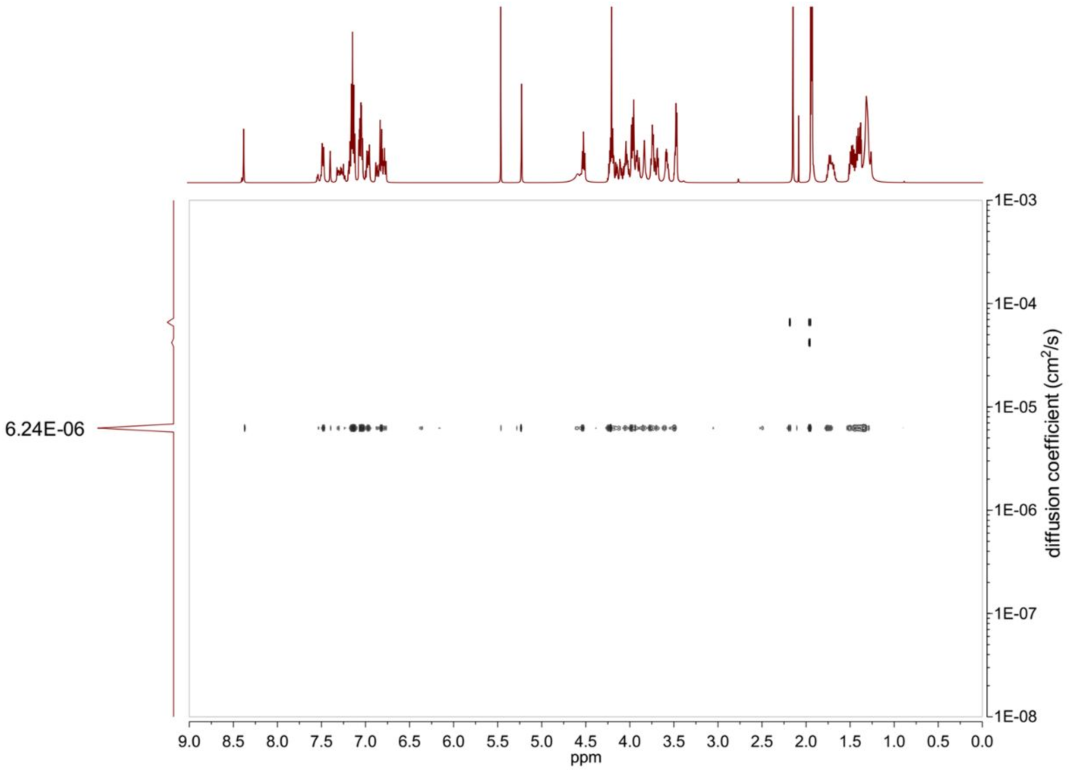

Figure S21. DOSY NMR spectrum (600 MHz, CD_3CN) of compound **DTM/C**.

References

(S1) Sumi, T.; Goseki, R.; Otsuka, H. Tetraarylsuccinonitriles as Mechanochromophores to Generate Highly Stable Luminescent Carbon-Centered Radicals. *Chem. Commun.* **2017**, *53*, 11885-11888.

(S2) Cuc, T. T. K.; Hung, C.-H.; Wu, T.-C.; Nhien, P. Q.; Khang, T. M.; Hue, B. T. B.; Chuang, W.-T.; Lin, H.-C. Force-Activated Ratiometric Fluorescence Switching of Tensile Mechano-Fluorophoric Polyurethane Elastomers with Enhanced Toughnesses Improved by Mechanically Interlocked [c2] Daisy Chain Rotaxanes. *Chem. Eng. J.* **2024**, *485*, 149694.

(S3) Khang, T. M.; Nhien, P. Q.; Cuc, T. T. K.; Wu, C.-H.; Hue, B. T. B.; Wu, J. I.; Li, Y.-K.; Lin, H.-C. Dual and Sequential Locked/Unlocked Photo-Switching Effects on FRET Processes by Tightened/Loosened Nano-Loops of Diarylethene-Based [1]Rotaxanes. *Chem. Commun.* **2023**, *59*, 466-469.

(S4) Cuc, T. T. K.; Lai, Y.-C.; Khang, T. M.; Chuang, W.-T.; Wu, T.-K.; Rotomskis, R.; Steponkiene, S.; Lin, H.-C. Effective Sliding Motions of Vibration-Induced Emission Stoppers in Mechanically Interlocked Molecules as Artificial Muscle Tougheners and In-Situ Molecular Shuttling Sensors for Self-Healable Mechano-Fluorescent Polyurethane Organogels. *Adv. Funct. Mater.* **2025**, e19737.

(S5) Wang, Z.; Ma, Z.; Wang, Y.; Xu, Z.; Luo, Y.; Wei, Y.; Jia, X. A Novel Mechanochromic and Photochromic Polymer Film: When Rhodamine Joins Polyurethane. *Adv. Mater.* **2015**, *27*, 6469-6474.

(S6) Khang, T. M.; Huang, R.; Khan, A.; Chuang, W.-T.; Nhien, P. Q.; Cuc, T. T. K.; Hue, B. T. B.; Wei, K.-H.; Li, Y.-K.; Lin, H.-C. Reversible Ratiometric Mechanochromic Fluorescence Switching in Highly Stretchable Polyurethane Elastomers with Ultratoughness Enhanced by Polyrotaxane. *ACS Materials Lett.* **2022**, *4*, 2537-2546.

(S7) Qin, J.; Chen, Y.; Guo, X.; Huang, Y.; Chen, G.; Zhang, Q.; He, G.; Zhu, S.; Ruan, X.; Zhu, H. Regulation of Hard Segment Cluster Structures for High-performance Poly(urethane-urea) Elastomers. *Adv. Sci.* **2024**, *11*, 2400255.

(S8) Wang, X.; Xu, J.; Zhang, Y.; Wang, T.; Wang, Q.; Li, S.; Yang, Z.; Zhang, X. A Stretchable, Mechanically Robust Polymer Exhibiting Shape-Memory-Assisted Self-Healing and Clustering-Triggered Emission. *Nat. Commun.* **2023**, *14*, 4712.

(S9) Zhu, Y.; He, Y.; Lu, W.; Tian, H.; Fei, F.; Zhou, P.; Wang, J. Multi-Functional Self-Healing Polyurethane Elastomer Based on Chair Conformation for Strain Sensors. *J. Mater. Chem. A* **2024**, *12*, 28716-28730.

(S10) Huang, Y.; Wu, H.; Li, W.; Yuan, Z.; Wu, Q.; Li, R.; Wu, J. A Healable Poly(urethane-urea) Elastomer with Ultra-High Mechanical Strength Enabled by Tailoring Multiple Relaxation Dynamics of Hierarchical Hard Domains. *J. Mater. Chem. A* **2022**, *10*, 24290-24300.

(S11) Xue, Y.; Lin, J.; Wan, T.; Luo, Y.; Ma, Z.; Zhou, Y.; Tuten, B. T.; Zhang, M.; Tao, X.; Song, P. Stretchable, Ultratough, and Intrinsically Self-Extinguishing Elastomers with Desirable Recyclability. *Adv. Sci.* **2023**, *10*, 2207268.

(S12) Fan, X.; Zhang, L.; Dong, F.; Liu, H.; Xu, X. Room-Temperature Self-Healing Polyurethane-Cellulose Nanocrystal Composites with Strong Strength and Toughness Based on Dynamic Bonds. *Carbohydr. Polym.* **2023**, *308*, 120654.

(S13) Guo, R.; Zhang, Q.; Wu, Y.; Chen, H.; Liu, Y.; Wang, J.; Duan, X.; Chen, Q.; Ge, Z.; Zhang, Y. Extremely Strong and Tough Biodegradable Poly(urethane) Elastomers with Unprecedented Crack Tolerance via Hierarchical Hydrogen-Bonding Interactions. *Adv. Mater.* **2023**, *35*, 2212130.

(S14) Zhu, X.; Zhang, W.; Lu, G.; Zhao, H.; Wang, L. Ultrahigh Mechanical Strength and Robust Room-Temperature Self-Healing Properties of a Polyurethane-Graphene Oxide Network Resulting from Multiple Dynamic Bonds. *ACS Nano* **2022**, *16*, 16724-16735.

(S15) Zhu, X.; Hao, Y.; Huang, L.-F.; Zhao, H.; Wang, L. Ultra-Robust, Highly Stretchable and Ambient Temperature Rapid Self-Healing Polyurethane/Graphene Elastomers Enabled by Multi-Type Hydrogen Bonds. *J. Mater. Chem. A* **2024**, *12*, 26158-26169.

(S16) Rong, H.; Zhang, Z.; Zhang, Y.; Lu, X. Self-Healing Elastomers with Unprecedented Ultrahigh Strength, Superhigh Fracture Energy, Excellent Puncture Resistance, and Durability Based on Supramolecule Interlocking Networks Formed by Interlaced Hydrogen Bonds. *ACS Appl. Mater. Interfaces* **2024**, *16*, 2802-2813.
